# Neural mechanisms supporting emotional and self-referential information processing and encoding in older and younger adults

**DOI:** 10.1093/scan/nsaa052

**Published:** 2020-04-17

**Authors:** Ryan T Daley, Holly J Bowen, Eric C Fields, Katelyn R Parisi, Angela Gutchess, Elizabeth A Kensinger

**Affiliations:** 1 Department of Psychology and Neuroscience, Boston College, Chestnut Hill, MA 02467, USA; 2 Department of Psychology, Southern Methodist University, Dallas, TX 75206, USA; 3 Department of Psychology, Brandeis University, Waltham, MA 02453, USA

**Keywords:** emotion, self-referencing, aging, memory

## Abstract

Emotion and self-referential information can both enhance memory, but whether they do so via common mechanisms across the adult lifespan remains underexplored. To address this gap, the current study directly compared, within the same fMRI paradigm, the encoding of emotionally salient and self-referential information in older adults and younger adults. Behavioral results replicated the typical patterns of better memory for emotional than neutral information and for self-referential than non-self-referential materials; these memory enhancements were present for younger and older adults. In neural activity, young and older adults showed similar modulation by emotion, but there were substantial age differences in the way self-referential processing affected neural recruitment. Contrary to our hypothesis, we found little evidence for overlap in the neural mechanisms engaged for emotional and self-referential processing. These results reveal that—just as in cognitive domains—older adults can show similar performance to younger adults in socioemotional domains even though the two age groups engage distinct neural mechanisms. These findings demonstrate the need for future research delving into the neural mechanisms supporting older adults’ memory benefits for socioemotional material.

## Introduction

Each moment, more information bombards us than we can process and remember. With age, prioritizing information becomes more difficult; relevant content is missed and irrelevant details are retained (Hasher *et al*., 1991; Kane *et al*., 1994). This pattern arises from age-related changes in the integrity of the lateral prefrontal cortex (Tisserand *et al*., 2002; Fjell *et al*., 2014) and the recruitment of attentional networks (Milham *et al*., 2002; Clapp *et al*., 2011; Spreng *et al*., 2016).

Emotional information is prioritized for processing (Eastwood *et al*., 2001; Vuilleumier and Schwartz, 2001; Kousta *et al*., 2009), as is information that is self-referential or relates to one’s identity or goals (Rogers *et al*., 1977; Symons and Johnson, 1997; Alexopoulos *et al*., 2012; Sui *et al*., 2012; Yin *et al*., 2019). This information remains well-prioritized with age, despite age-related cognitive declines. Older and younger adults show better memory for emotional compared to neutral stimuli (for review: Kensinger, 2009) and for information encoded in relation to the self rather than another person (Gutchess *et al*., 2007). Memory performance of older adults can match the level of younger adults for both emotional and self-referential content, despite general deficits in episodic memory that occur with aging (discussed in Kensinger and Gutchess, 2017).

The current study tests two predictions that arose from this relative age preservation of memory enhancements for emotional and self-referential processing. First, emotional and self-referential information will be attended to and remembered using a different circuitry than is used for neutral and non-self-referential information. If correct, there should be differences in the brain regions related to the processing and successful encoding of emotional *vs* neutral information and of self-referential *vs* non-self-referential information. Moreover, this different circuitry may be relatively spared in aging (Kensinger and Gutchess, 2017), leading to age stability in patterns of activation. Across the adult lifespan, the encoding of emotional content is associated with the engagement of limbic structures including the amygdala, orbitofrontal cortex, anterior cingulate cortex and the striatum, as well as regions implicated in the default mode network, including the medial prefrontal cortex (mPFC), posterior cingulate, precuneus and angular gyri (Kensinger and Leclerc, 2009; Mather, 2016). The processing and successful encoding of self-referential content is strongly associated with activity in the mPFC and other cortical midline structures, including the precuneus and posterior cingulate cortex, as well as a network extending to lateral temporal and parietal regions (Macrae *et al*., 2004; Turk *et al*., 2011; Kim and Johnson, 2014; Morel *et al*., 2014; Kalenzaga *et al*., 2015). Second, emotional and self-referential material may be processed and remembered using overlapping mechanisms. Gutchess and Kensinger (2018) proposed that—at least when memory is tested after relatively short delays—participants may remember self-referential material well for many of the same reasons that they remember emotional information well, including enhanced attention and prioritized processing. If so, there should be substantial overlap in the brain regions engaged for emotional *vs* neutral information and for self-referential *vs* non-self-referential information. While there are a number of regions involved in self-referencing and emotional processing, one of the common regions identified across studies is the mPFC. Thus, we hypothesized that processing and encoding these types of content would converge in this region.

This shared-mechanism hypothesis converges with a recent study in which older adults encountered emotional and neutral vignettes under three encoding conditions: syllable counting (baseline), semantic elaboration and self-referencing (Grilli *et al*., 2018). Memory was better for vignettes presented in the self-referencing condition compared to baseline and semantic elaboration. There were also emotional memory enhancements for baseline and semantic elaboration, but not for self-referencing. This pattern would be expected if overlapping mechanisms supported emotional and self-referential memory enhancements (Gutchess and Kensinger, 2018): The presence of both dimensions does not necessarily benefit beyond the presence of a single dimension, because memory modulation is already triggered.

Although we predicted emotional and self-referential information would be processed via overlapping mechanisms that were (i) distinct from those engaged for neutral or non-self-referential material and (ii) relatively preserved with age, there are alternative outcomes. First, older adults sometimes reach the same behavioral outcome as younger adults via alternate neural means. During challenging cognitive tasks, older adults increase brain activity in regions not recruited by younger adults; the relation of this recruitment to performance suggests compensation for reduced activity in other regions (Gutchess *et al*., 2005; Reuter-Lorenz and Cappell, 2008; Cabeza *et al*., 2018). Older adults compensatorily recruit prefrontal cortex bilaterally when younger adults recruit regions unilaterally (Cabeza *et al*., 2002; Dolcos *et al*., 2002). Older adults also shift from posterior to anterior cortical activation during episodic memory retrieval compared to younger adults (Davis *et al*., 2008). Although there is little research on these differences in the socioemotional memory literature (St. Jacques *et al*., 2009), it is plausible that older adults show relatively preserved memory for emotional and self-referential material not because that circuitry is preserved but because they are able to recruit different, potentially compensatory, mechanisms.

Second, while we predicted overlap in the mechanisms supporting the processing and encoding of emotional and self-referential information, divergence was also possible. The memory enhancements for emotional and self-referential information are commonly described as independent spheres of influence. Some, but not all, prior research lends support to the overlapping-mechanism hypothesis. For instance, one ERP study (Fields and Kuperberg, 2012) yielded results consistent with a shared mechanism, with larger amplitude in the late positive time window, a marker of sustained attention (Citron, 2012), for emotional information compared to neutral information. This increase in amplitude was comparable to that of self-referential content, regardless of emotion, suggesting a common mechanism may contribute to enhanced processing of both self-referential and emotional information. However, not every study has shown this pattern, as several demonstrate the combination self-referencing and emotion drive enhanced late positivities (Herbert *et al.*, 2011a,b; Schindler *et al*., 2014; Pinheiro *et al*., 2016; Fields and Kuperberg, 2016) or prolonged early posterior negativity (Bayer *et al*., 2017).

To test our predictions, the current study utilized a new paradigm in which young and older adults encoded emotionally salient or neutral objects within a self-referential or non-self-referential frame. We expected to replicate the typical behavioral patterns of better memory for emotional than neutral information and better memory for self-referential than non-self-referential materials, for both younger and older adults. We additionally examined whether emotion and self-referencing interacted to influence memory performance. For neural activity, we asked two key questions. First, would there be age similarity in processing emotional and self-referential information? Second, would similar regions be engaged for emotional and self-referential information, or would these two categories of information diverge in their neural processing?

## Methods

### Participants

Younger adults (*n* = 59; 29 female) ages 18–39 and older adults (*n* = 47; 33 female) ages 60–88 are included in behavioral analyses. The fMRI subsample consisted of 45 younger (23 female) and 35 older adults (23 female). Supplementary Materials detail exclusions and eligibility criteria. Table 1 displays performance on cognitive tests (see Supplementary Materials). Participants completed informed consent forms approved by the Boston College Institutional Review Board.

**Table 1:** Cognitive performance (mean, standard error) is reported for older and younger adult participants, and significance of group differences is included (*P*-value column)

	Older adults		Younger adults		df	*t*	*P*	
	*M*	SE	*M*	SE				
Digit Symbol	55.50	1.65	74.39	1.40	93.59	−8.73	<0.001	^***^
CVLT Short Delay Free Recall	12.57	0.38	14.16	0.30	85.25	−3.26	0.002	^**^
CVLT Long Delay Free Recall	13.11	0.36	14.02	0.30	86.21	−1.94	0.055	
Digit Comparison	61.57	1.28	82.96	1.83	92.28	−9.56	<0.001	^***^
Digits Backward	8.48	0.39	8.40	0.34	87.56	0.15	0.881	
Digits Forward	11.52	0.37	12.09	0.36	89.00	−1.09	0.277	
FAS	45.84	1.60	44.49	1.54	86.80	0.61	0.545	
Verbal Paired Associates I	24.13	1.02	26.43	0.80	83.97	−1.78	0.079	
Verbal Paired Associates II	7.04	0.29	7.91	0.04	47.01	−2.92	0.005	^**^
Visual Paired Associates I	15.35	0.46	17.20	0.24	67.79	−3.56	<0.001	^***^
Visual Paired Associates II	5.72	0.11	6.00	0.00	45.00	−2.66	0.011	^*^
Logical Memory I	29.54	0.91	27.98	0.87	88.90	1.24	0.217	
Logical Memory II	31.76	1.21	30.67	1.05	87.58	0.68	0.496	
Mental Control	25.22	0.76	27.80	0.82	88.27	−2.31	0.023	^*^
Mental Arithmetic	16.15	0.39	15.58	0.50	83.88	0.90	0.369	
Shipley Vocabulary Test	36.42	0.47	32.31	0.76	91.27	4.59	<0.001	^***^

^*^ *P* < 0.05.

^**^ *P* < 0.01.

^***^ *P* < 0.001.

Note: Fifteen younger adults had missing data for Verbal Paired Associates. Fourteen younger adults had missing data for CVLT, Digit Span, F-A-S, Mental Arithmetic, Mental Control, Logical Memory and Visual Paired Associates. Three younger adults had missing data for Digit Symbol and Digit Comparison, and two younger adults were missing data for the Shipley Vocabulary Test. Five older adults had missing data for Digit Comparison, three older adults had data missing for F-A-S, two older adults had data missing for the Shipley Vocabulary Test, and one older adult had data missing for CVLT, Digit Span, Digit Symbol, Mental Control, Mental Arithmetic, Logical Memory, Verbal Paired Associates and Visual Paired Associates. (See supplemental materials for more information about the cognitive testing.)

### Materials

#### Experimental stimuli

Stimuli included 420 images of objects selected from the Open Affective Standardized Image Set (OASIS; Kurdi *et al*., 2017) and image sets from prior research (e.g. Waring and Kensinger, 2009). See Supplementary Materials for norming details and Table 2 for participant ratings. The final stimulus set contained 140 objects of each emotional valence (negative, neutral and positive).

**Table 2:** Valence, arousal and self-relevance stimuli ratings (mean, standard error)

	Normative data		Older adults		Younger adults	
	*M*	SE	*M*	SE	*M*	SE
Valence						
Negative	3.67	0.07	4.61	0.06	4.39	0.07
Neutral	5.02	0.03	5.45	0.05	5.31	0.04
Positive	6.39	0.05	6.37	0.10	6.23	0.07
Arousal						
Negative	5.03	0.12	5.54	0.08	5.52	0.07
Neutral	4.02	0.07	5.10	0.07	5.06	0.04
Positive	4.84	0.06	5.30	0.15	5.47	0.09
Self-relevance						
Negative	3.99	0.10	−	−	−	−
Neutral	4.07	0.09	−	−	−	−
Positive	4.13	0.08	−	−	−	−

Note: Participants in the experiment were not asked to rate the self-relevance of stimuli as we expected that the condition in which they had the stimuli studied (i.e. self or other) would affect their later ratings.

### Procedures

**Fig. 1:**
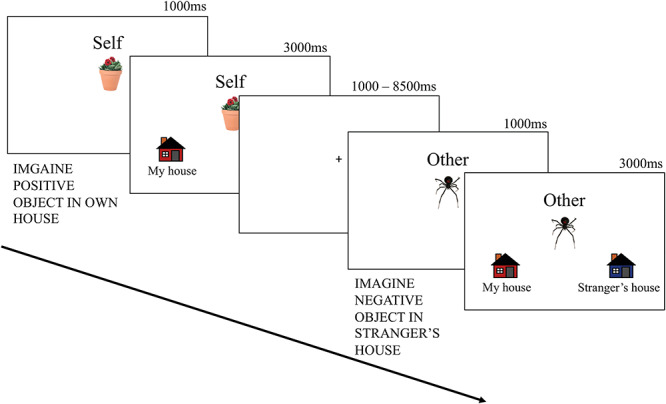
Full attention encoding. Participants were presented with positive, negative and neutral objects and asked to imagine placing them into the house corresponding to the word at the top of the screen (1000 ms). When the houses appeared at the bottom of the screen, participants were instructed to make a button press (1 = ‘Self’; 2 = ‘Stranger’) to indicate the appropriate house in which they imagined placing the object (3000 ms).

Participants received task instructions and practice trials prior to entering the MRI scanner. During the task, participants viewed images of negative, neutral and positive objects (Figure 1). Upon presentation of each object, a word appeared at the top of the screen (‘Self’ or ‘Other’). Participants were instructed to imagine the objects in either their own home (‘Self’) or in a stranger’s home (‘Other’). After 1000 ms, pictures of two houses appeared below the object (‘My House’ and ‘Stranger’s House’), and participants pressed a button indicating the house in which they imagined the object; participants had 3000 ms to complete each trial. For example, if participants viewed a picture of a flower with the word ‘Self’ above, they were to imagine the flower in their own house or yard; this was intended as a way for them to take ownership of the object. In contrast, if ‘Other’ appeared above the picture, they imagined the object in a stranger’s dwelling. They then pressed the appropriate button.

The task consisted of four full and two divided attention runs (*n* = 42 objects per run). Participants were presented with an equal number of ‘Self’ and ‘Other’ cues (*n* = 21 objects per condition per run). Each condition had equal numbers of negative, neutral and positive objects (*n* = 7 per valence per run). Five encoding sets were created in order to balance, across participants, whether objects appeared in the self full attention (28 per valence), other full attention (28 per valence), self divided attention (14 per valence), other divided attention (14 per valence) or were held out as lures (56 per valence). All participants completed the same recognition test, regardless of their encoding set, which included all 252 encoded items along with 168 new objects. As it was not the intent to examine trial-level fMRI responses to the divided attention trials (these were included as part of a larger study examining the automaticity of encoding of emotional and self-referential information), these trials were not included in behavioral or fMRI analyses in the current study and are not discussed further.

After a delay of approximately 30 minutes, participants completed an unexpected, self-paced recognition memory task. Participants completed practice trials and then made old/new judgements for each object using a computer keyboard. If participants indicated ‘new’, the next trial appeared. If participants indicated ‘old’, they were asked to make a remember/know/guess judgment (adapted from Rajaram, 1993). ‘Remember’ responses indicated that participants remembered specific object details. ‘Know’ responses indicated that participants knew they saw the object, but did not recall specific details. ‘Guess’ responses reflected no knowledge of study history. After completion of the memory task, participants viewed all objects again and made valence and arousal ratings.

Encoding stimuli were presented with E-Prime 2.0 (Psychology Software Tools, Inc., Pittsburgh, PA, USA) and viewed using a mirror mounted on the head coil. Responses were collected using a MR-compatible button box. All stimuli presented during the memory task and valence and arousal ratings were presented with PsyScope X B57 (International School of Advanced Studies, Trieste, Italy).

#### Scoring of memory data

D-prime scores (Macmillan *et al*., 2004) were calculated. Because lure items could not be assigned to the self-referential condition (i.e. items only become self-referential through the encoding manipulation), the emotion and neutral false alarm rates were used to calculate d-prime scores. For example, the d-prime_emotion_self_ score was calculated using the following equation: *z*[hit rate_emotion_self_] − *z*[false alarm rate_emotion_], and the d-prime_emotion_other_ score was calculated similarly. D-prime scores were subjected to a repeated measures ANOVA, with emotionality (emotion/neutral) and self-referencing (self/other) as within-subject factors and age group (older/younger adults) as a between-subject factor (note: ‘Know’ responses were excluded from the behavioral analyses and not included in the fixed effects fMRI models due to low response rates; for analyses of ‘Know’ responses, see Supplementary Figure S1).

#### FMRI image acquisition

Data were collected on a Siemens Magnetom Prisma^fit^ scanner with a 32-channel head coil. Functional images were acquired using a simultaneous multi-slice EPI sequence (Coronal Slices = 69, Voxel Size = 2 mm^3^, FOV = 208 mm, TR = 2500 ms, TE = 28 ms, Flip Angle = 75°, Base Resolution = 104, Echo Spacing = 0.67 ms).

#### FMRI image preprocessing

All fMRI data were preprocessed and analyzed using SPM12 (Wellcome Department of Cognitive Neurology, London, UK) via MATLAB version R2016a (The Mathworks Inc.). Structural and functional images were reoriented to the anterior commissure. Functional scans were realigned and unwarped to provide motion correction, with all images set to match the mean image, co-registered to the structural scan and normalized to the MNI template (written at 2 mm voxels) using a two-step process that first segmented and normalized the structural scans and then applied those normalization parameters to the functional images. Functional images were also smoothed with a full-width at half maximum 6mm^3^ Gaussian kernel. Participants were excluded if their linear motion parameters (x, y, z) or rotational motion parameters (pitch, roll, yaw) extended beyond ±5 mm or 3°, respectively.

Two general linear models were created to assess neural activity during the encoding task, using a subsequent memory event-related design. Each trial was modeled as an event (duration = 0). In both models, old remembered objects were compared to forgotten (new or guess response) objects. To be included, participants had to have at least five trials in each of these bins.

To compare emotional *vs* neutral processing, each participant’s data were subjected to a fixed effects model collapsing across self-referential conditions and consisting of regressors for emotional remembered, emotional forgotten, neutral remembered and neutral forgotten (see Supplementary Data for both behavioral and neural results broken down by emotional valence). ‘Known’ objects were modeled as a regressor of no interest, along with a linear drift regressor. These results were then brought to a second-level, random-effects ANOVA using emotion/neutral and remembered/forgotten as within-subject variables and age as a between-subject variable. This ANOVA will be referred to as the ‘emotion/neutral’ ANOVA. When significant interactions were revealed, post hoc analyses were conducted to determine the direction of the interaction.

To examine neural activity associated with self-referencing, each participant’s data were collapsed across emotional valence and subjected to a first-level model consisting of regressors for self remembered, self forgotten, other remembered and other forgotten. ‘Known’ objects were modeled as a regressor of no interest, along with a linear drift regressor. The results from this model were then subjected to a second-level, random-effects ANOVA using self/other and remembered/forgetten as within-subject variables and age as a between-subject variable. This ANOVA will be referred to as the ‘self/other’ ANOVA. When significant interactions were revealed, post hoc analyses were conducted to determine the direction of the interaction.

To visualize the overlap, or lack thereof, in the emotion/neutral and self/other ANOVAs, conjunction analyses were used to overlay the activation from the two models.

#### FMRI data visualization

FMRI renderings and any discussed clusters reveal effects that survive their respective *F*-test at *P* < 0.005 and a voxel extent of *k* = 40 contiguous voxels [Monte Carlo simulations were used to determine this voxel extent to correct for multiple comparisons at *P* < 0.05; Slotnick *et al*., 2003; Slotnick, 2017]. Renderings are color-coded according to directional *t*-contrasts (e.g. to distinguish a main effect of emotion that reflects emotion > neutral from one that reflects neutral > emotion). Tables report all results with a voxel extent of *k* > 10, to avoid Type II error, should future meta-analyses be conducted using these data. All coordinates derived from SPM12 were converted to Talairach coordinates using the GingerALE (http://www.brainmap.org/ale/) icbm_spm2tal transform and manually checked with the Talairach atlas (Talairach and Tournoux, 1988).

## Results

### Behavioral performance

**Fig. 2:**
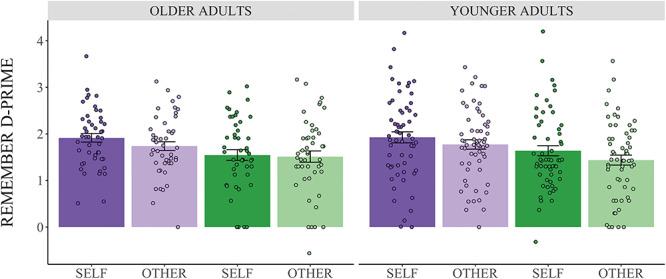
Behavioral memory performance. Error bars represent ± SEM. There was a main effect of emotionality. Both groups had better memory for emotional objects (purple bars) compared to neutral objects (green bars). There was also a main effect of self-relevance. Both groups had better memory for objects in the self condition (dark bars) compared to objects in the other condition (light bars).

The ANOVA of d-prime scores revealed a main effect of emotionality [*F*(1,104) = 43.59, *P* < 0.001, ${\upomega}_G^2$*=* 0.034 (Olejnik and Algina, 2003)], such that, across groups, memory was better for emotional than for neutral objects (Figure 2). Similarly, there was a main effect of self-referencing [*F*(1,104) = 26.05, *P <* 0.001, ${\upomega}_G^2$ = 0.008], such that across groups, memory for objects was better in the self than other condition. Interestingly, there was no main effect of age group [*F*(1,104) = 0.01, *P* = 0.92, ${\upomega}_G^2$ = −0.008], and age did not interact with emotionality [*F*(1,104) = 0.03, *P* = 0.87, ${\upomega}_G^2$ = −0.001] or self-referencing [*F*(1,104) = 1.61, *P* = 0.21, ${\upomega}_G^2$ = 0.000]. Thus young and older adults’ performance on the task, and their memory benefits from emotion and self-referencing, did not significantly differ. Emotionality did not interact with self-referencing [*F*(1,104) = 0.87, *P* = 0.35, ${\upomega}_G^2$ = 0.000], and there was no three-way interaction between emotional valence, self-referencing and age [*F*(1,104) = 3.49, *P* = 0.07, ${\upomega}_G^2$ = 0.001].

### Imaging results

#### Emotion effects

**Fig. 3:**
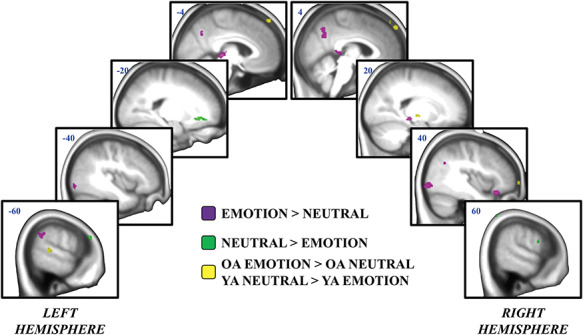
Processing emotional and neutral information. *n* = 77 (older adults = 34; younger adults = 43). One older adult and two younger adults were excluded from the emotion/neutral ANOVA due to small bin sizes in their fixed effects models. All clusters represent activity at the whole-brain group level. Activity associated with the processing of emotional > neutral stimuli is depicted in purple. Activity associated with the processing of neutral > emotional stimuli is depicted in green. The yellow regions depict an age by emotion interaction (‘OA’ = older adults; ‘YA’ = younger adults).

The emotion/neutral ANOVA primarily revealed clusters showing a significant main effect of emotion, with no emotion-by-age interaction (Figure 3 and Table 3). Directional *t*-tests revealed that across older and younger adults, posterior regions were primarily engaged for emotion > neutral information. This included activation in bilateral inferior occipital gyri and other portions of the ventral visual stream, including right inferior temporal gyrus. Participants also engaged the precuneus, left supramarginal gyrus and bilateral angular gyri, left thalamus, left lingual gyrus and right hippocampus. Although there was some activity in the right lateral orbitofrontal cortex that was greater for emotional than neutral information, on the whole, there was greater activation for neutral > emotional information in anterior regions. This included the anterior cingulate, right precentral gyrus and right inferior frontal gyrus.

**Table 3:** Group-level coordinates during the processing of emotional and neutral information

Lobe	Hemisphere	Region	BA	MNI (*x*, *y*, *z*)	Tal (*x*, *y*, *z*)	Cluster extent	*P* < 0.005, *k* = 40	Direction
Emotion > neutral								
Frontal	Right	Inferior frontal gyrus	9	58, 28, 18	53, 22, 24	30		
Frontal	Left	Inferior frontal gyrus	45	−50, 24, 4	−47, 20, 9	32		
Frontal	Right	Lateral orbitofrontal gyrus	47	38, 26, −20	34, 24, −11	71	Yes	
Frontal	Right	Lateral orbitofrontal gyrus	47	50, 30, −4	45, 26, 4	37		
Frontal	Left	Lateral orbitofrontal gyrus	47	−48, 22, −6	−45, 19, 0	16		
Frontal	Left	Middle frontal gyrus	6	−44, 8, 54	−42, 1, 52	13		
Temporal	Left	Fusiform gyrus	37	−36, −44, −22	−34, −41, −20	35		
Temporal	Right	Fusiform gyrus	20	40, −44, −20	36, −41, −17	22		
Temporal	Left	Fusiform gyrus	37	−42, −54, −18	−40, −50, −17	23		
Temporal	Right	Hippocampus	27	18, −28, −8	16, −27, −5	54	Yes	
Temporal	Right	Inferior temporal gyrus	20	50, −14, −38	46, −12, −30	62	Yes	
Temporal	Left	Middle temporal gyrus	22	−58, −44, 6	−55, −43, 5	12		
Parietal	Right	Angular gyrus	39	52,−58, 30	47, −59, 27	91	Yes	
Parietal	Right	Precuneus	31	6, −62, 32	4, −62, 27	151	Yes	
Parietal	Right	Precuneus posterior cingulate	7/31	14, −52, 34	11, −53, 30	15		
Parietal	Left	Supramarginal gyrus, angular gyrus	39/44	−58, −48, 32	−55, −49, 28	137	Yes	
Occipital	Left	Fusiform gyrus	37	−42, −72, −16	−40, −67, −17	31		
Occipital	Right	Fusiform gyrus	37	42, −62, −18	38, −58, −17	36		
Occipital	Right	Inferior occipital gyrus	18	36, −88, −8	32, −83, −10	170	Yes	
Occipital	Left	Inferior occipital gyrus	18	−36, −86, −8	−34, −81, −11	120	Yes	
Other	Right	Brainstem	N/A	6, −20, −20	5, −19, −15	14		
Other	Right	Cerebellum	N/A	16, −36, −44	14, −32, −38	31		
Other	Left	Cerebellum	N/A	−4, −40, 2	−5, −39, 2	10		
Other	Left	Fornix	N/A	2, 0, −6	1, −1, −1	13		
Other	Left	Hypothalamus	N/A	−12, −6, −8	−12, −7, −4	20		
Other	Left	Posterior cingulate	29/31	−8, −48, 22	−9, −48, 19	10		
Other	Left	Posterior cingulate	31	−14, −44, 34	−14, −46, 30	10		
Other	Left	Thalamus lingual gyrus	N/A	−4, −30, 0	−5, −30, 1	171	Yes	
Neutral > emotion								
Frontal	Right	Middle frontal gyrus	6	24, 30, 16	21, 25, 21	13		
Frontal	Right	Precentral gyrus	6	32, 0, 26	28, −4, 28	46	Yes	
Frontal	Right	Precentral gyrus inferior frontal gyrus	6/44	54, 2, 20	49, −2, 23	96	Yes	
Temporal	Right	Superior temporal gyrus	42	56, −2, 2	51, −4, 7	12		
Parietal	Right	Inferior parietal lobule	40	48, −28, 38	43, −31, 36	19		
Other	Left	Anterior cingulate	24/32	−16, 28, −10	−16, 25, −3	99	Yes	
Other	Left	Anterior cingulate	32	0, 10, −12	−1, 9, −6	23		
Other	Right	Anterior cingulate	32	20, 22, 30	17, 16, 33	17		
Other	Left	Corpus callosum	N/A	−12, −12, 28	−12, −15, 28	22		
Other	Right	Dorsal anterior cingulate	24	14, 2, 46	11, −4, 46	24		
Emotion/neutral × age interaction								
Frontal	Right	Postcentral gyrus	43	56, −8, 26	50, −12, 27	11		YA neutral > YA emotion
Frontal	Left	Precentral gyrus	4	−58, −6, 26	−55, −9, 26	38		YA neutral > YA emotion
Frontal	Right	Precentral gyrus	6	64, −4, 10	58, −7, 14	17		YA neutral > YA emotion
Temporal	Left	Inferior temporal gyrus	37	−64, −48, −14	−60, −45, −14	17		n.s.
Temporal	Left	Middle temporal gyrus	21	−62, −36, 6	−59, −36, 5	76	Yes	OA emotion > OA neutral
Temporal	Left	Middle temporal gyrus	21	−42, −56, 8	−40, −54, 6	12		n.s.
Temporal	Right	Parahippocampal gyrus	36	28, −20, −32	25, −18, −26	16		OA emotion > OA neutral
Temporal	Right	Superior temporal gyrus	22	40, −30, −2	36, −30, 0	14		n.s.
Temporal	Right	Superior temporal gyrus	22	68, −14, −4	62, −15, 0	28		YA neutral > YA emotion
Temporal	Left	Superior temporal gyrus, middle temporal gyrus	21/22	−58, −18, 0	−55, −18, 2	22		YA neutral > YA emotion
Parietal	Left	Superior parietal lobule	7	−38, −62, 64	−37, −65, 55	32		YA neutral > YA emotion
Occipital	Left	Middle occipital gyrus, inferior temporal gyrus	19/37	−48, −62, −4	−46, −59, −6	26		n.s.
Other	Left	Cerebellum	N/A	−52, −44, −32	−49, −40, −29	17		OA emotion > OA neutral
Other	Right	Cerebellum	N/A	4, −88, −38	3, −80, −38	29		YA neutral > YA emotion
Other	Right	Cerebellum	N/A	22, −52, −30	20, −48, −27	14		YA neutral > YA emotion
Other	Right	Cerebellum	N/A	6, −84, −46	5, −76, −44	13		OA emotion > OA neutral
Other	Right	Basal ganglia thalamus	N/A	18, −14, −4	16, −15, 0	59	Yes	OA emotion > OA neutral
Other	Right	Posterior cingulate cortex	26	6, −44, 14	4, −44, 13	13		OA emotion > OA neutral

Note: n.s. in the ‘Direction’ column indicates significant clusters for the overall emotion/neutral × age interaction that were not significant in the contrasts of interest (i.e. OA emotion > OA neutral; YA neutral > YA emotion).

There was a significant emotion-by-age interaction in a small number of regions, within the left middle temporal gyrus and right basal ganglia and thalamus. To determine the direction of these interactions, post hoc directional interaction *t*-tests were conducted. Only one direction of the interaction reached significance (greater emotion > neutral effect for older adults than younger adults): All three regions were engaged more by older adults for emotional > neutral information. No regions were engaged more by younger adults for neutral > emotional information.

#### Self-referencing effects

**Fig. 4:**
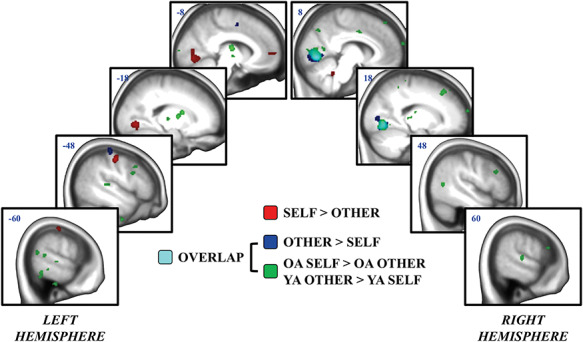
Processing self-relevant and non-self-relevant information. *n* = 80 (older adults = 35; younger adults = 45). All clusters represent activity at the whole-brain group level. Activity associated with the processing of self > other stimuli is depicted in red. Activity associated with the processing of other > self stimuli is depicted in blue. The green regions depict an age by self-relevance interaction (‘OA’ = older adults; ‘YA’ = younger adults). The regions depicted in cyan represent overlapping clusters between the other > self and age by self-relevance interaction.

The self/other ANOVA also revealed many clusters that showed a significant and age-invariant main effect of self-referencing (Figure 4 and Table 4). Directional *t*-tests revealed that both age groups engaged the left ventromedial prefrontal cortex, left postcentral gyrus, left thalamus, left lingual gyrus and cerebellum when processing self > other information, while they engaged the right lingual gyrus, left postcentral gyrus and bilateral precentral gyri and bilateral putamen when processing other > self information.

**Table 4:** Group-level coordinates when processing self-relevant and non-self-relevant stimuli

Lobe	Hemisphere	Region	BA	MNI (*x*, *y*, *z*)	Tal (*x*, *y*, *z*)	Cluster extent	*P* < 0.005, *k* = 40	Direction
Self > other								
Frontal	Left	Ventromedial prefrontal cortex	10	−6, 54, 2	−6, 48, 11	54	Yes	
Temporal	Left	Superior temporal gyrus	38	−42, 12, −20	−40, 11, −13	14		
Parietal	Left	Lingual gyrus cerebellum	18	−12, −76, −6	−12, −72, −8	383	Yes	
Parietal	Left	Postcentral gyrus	1	−46, −16, 48	−44,−21, 45	240	Yes	
Other	Right	Cerebellum	N/A	54, −72,−32	49, −66, −30	49	Yes	
Other	Left	Cerebellum	N/A	−2, −42, −28	−3, −38, −25	95	Yes	
Other	Right	Cerebellum	N/A	36, −52,−40	33, −47, −36	10		
Other	Left	Basal ganglia	N/A	−2, −2, −6	−3, −3, −1	10		
Other	Left	Thalamus	N/A	2, −16, 4	1, −17, 6	51	Yes	
Other > self								
Frontal	Right	Precentral gyrus	6	66, 8, 14	60, 4, 18	20		
Frontal	Left	Precentral gyrus	6	−4, −4, 54	−5, −10, 52	71	Yes	
Frontal	Right	Precentral gyrus	4	46, 0, 16	41, −3, 19	15		
Frontal	Right	Precentral gyrus	6	34, −10, 64	30, −17, 61	69	Yes	
Temporal	Right	Hippocampus	34	22, −16, −10	19, −16, −6	22		
Temporal	Left	Inferior temporal gyrus	37	−66, −56, −14	−62, −52, −14	14		
Temporal	Right	Middle temporal gyrus	21	62, −40, −12	56, −38, −9	11		
Temporal	Right	Parahippocampal gyrus	28	22, −20, −20	20, −19, −15	18		
Temporal	Right	Superior temporal gyrus	42	68, −20, 8	62, −21, 10	17		
Parietal	Left	Inferior parietal lobule	7	−32, −52, 64	−32, −56, 56	19		
Parietal	Right	Postcentral gyrus	4	42, −24, 60	37, −30, 56	10		
Parietal	Left	Postcentral gyrus precentral gyrus	3/4/6	−42, −24, 60	−41, −29, 55	719	Yes	
Parietal	Left	Superior parietal lobule	7	−8, −68, 66	−9, −71, 57	10		
Parietal	Right	Superior parietal lobule	7	34, −70, 54	30, −72, 47	10		
Occipital	Left	Cuneus	18	−4, −106, 20	−5, −102, 13	22		
Occipital	Right	Lingual gyrus	18	12, −72, −4	10, −69, −6	870	Yes	
Other	Right	Pons	N/A	2,−24, −36	1,−21, −30	18		
Other	Left	Pons	N/A	−2, −14, −36	−2, −12, −29	10		
Other	Left	Putamen	N/A	−24, 4, 2	−23, 2, 6	142	Yes	
Other	Right	Putamen	N/A	24, −2, 6	21, −4, 10	57	Yes	
Other	Left	Putamen	N/A	−24, 0, −10	−23, −1, −5	15		
Other	Right	Putamen	N/A	28, 0, −8	25, −1, −3	15		
Self/other × age interaction								
Frontal	Right	Dorsomedial prefrontal cortex	6	6, 42, 56	4, 32, 58	43	Yes	n.s.
Frontal	Left	Dorsomedial prefrontal cortex	8	0, 24, 52	−2, 16, 53	54	Yes	OA self > OA other
Frontal	Left	Dorsomedial prefrontal cortex	6	−10, 12, 54	−11, 5, 53	10		OA self > OA other
Frontal	Left	Inferior frontal gyrus	44	−48, 12, 26	−46, 7, 28	68	Yes	YA other > YA self
Frontal	Right	Medial orbitofrontal cortex	11	4, 32, −24	3, 30, −14	10		n.s.
Frontal	Left	Middle frontal gyrus	10	−28, 62, −4	−27, 56, 6	186	Yes	OA self > OA other; YA other > YA self
Frontal	Right	Middle frontal gyrus	10	38, 62, 2	34, 56, 12	63	Yes	YA other > YA self
Frontal	Right	Middle frontal gyrus	9	52, 24, 28	47, 18, 32	98	Yes	n.s.
Frontal	Left	Middle frontal gyrus	6	−30, 14, 56	−29, 7, 55	21		n.s.
Frontal	Right	Middle frontal gyrus	8	44, 18, 30	39, 12, 33	10		YA other > YA self
Frontal	Right	Paracentral lobule	4	24, −42, 62	20, −46, 56	55	Yes	YA other > YA self
Frontal	Right	Precentral gyrus	4	8, −16, 74	5, −23, 69	36		YA other > YA self
Frontal	Right	Precentral gyrus	4	22, −24, 50	19, −29, 47	11		YA other > YA self
Frontal	NA	Precentral gyrus	6	−30, −6, 30	−29, −10, 30	18		n.s.
Frontal	Left	Precentral gyrus	4	−50, 2, 48	−48, −4, 46	27		YA other > YA self
Frontal	Right	Precentral gyrus	4	46, 16, 48	41, 9, 49	38		YA other > YA self
Frontal	Left	Precentral gyrus	6	−22, −4, 42	−22, −9, 41	26		n.s.
Frontal	Left	Precentral gyrus	4	−38, −20, 64	−37, −26, 59	38		YA other > YA self
Frontal	Right	Precentral gyrus, postcentral gyrus	1/3/4	30, −20, 70	26, −27, 65	133	Yes	YA other > YA self
Frontal	Left	Prefrontal gyrus	4	−54, −6, 46	−52, −11, 44	20		YA other > YA self
Frontal	Right	Superior frontal gyrus, middle frontal gyrus	6	18, 28, 48	15, 20, 50	89	Yes	OA self > OA other
Frontal	Right	Ventromedial prefrontal cortex	10	4, 46, −14	3, 42, −4	47	Yes	n.s.
Temporal	Left	Fusiform gyrus	36	−32, −44, −14	−31, −41, −13	11		n.s.
Temporal	Left	Hippocampus	28	−18, −6, −32	−17, −4, −25	21		OA self > OA other
Temporal	Left	Inferior temporal gyrus	20/37	−62, −50, −18	−58, −46, −17	136	Yes	YA other > YA self
Temporal	Right	Inferior temporal gyrus	20	60, −40, −18	55, −38, −15	13		n.s.
Temporal	Right	Middle temporal gyrus	37	46, −60, 8	41, −59, 7	64	Yes	OA self > OA other; YA other > YA self
Temporal	Left	Middle temporal gyrus	21	−58, −16, −8	−55, −16, −5	13		n.s.
Temporal	Right	Middle temporal gyrus	21	64, −44, −4	58, −43, −2	12		YA other > YA self
Temporal	Right	Middle temporal gyrus	21	60, −2, −32	55, −1, −24	23		n.s.
Temporal	Left	Middle temporal gyrus	21	−60, 6, −24	−56, 6, −18	13		n.s.
Temporal	Right	Middle temporal gyrus	21	56, −4, −20	51, −4, −13	10		YA other > YA self
Temporal	Right	Parahippocampal gyrus	35	30, −24, −22	27, −22, −17	13		OA self > OA other
Temporal	Right	Superior temporal gyrus	22/42	70, −28, 10	64, −29, 12	234	Yes	YA other > YA self
Temporal	Right	Superior temporal gyrus	20	60, 6, −12	55, 5, −5	18		n.s.
Temporal	Left	Superior temporal gyrus	22/39/42	−64, −42, 10	−60, −41, 8	283	Yes	YA other > YA self
Temporal	Left	Superior temporal gyrus	38	−46, 10, −24	−43, 10, −17	20		n.s.
Temporal	Right	Superior temporal gyrus	38	46, 22, −34	42, 21, −24	24		OA self > OA other
Temporal	Right	Superior temporal gyrus	36	32, −6, −40	29, −4, −32	11		n.s.
Temporal	Left	Superior temporal gyrus, transverse temporal gyrus	41/42	−48, −34, 8	−46, −34, 8	56	Yes	YA other > YA self
Temporal	Right	Transverse temporal gyrus	41	40, −36, 8	36, −36, 9	15		OA self > OA other
Parietal	Right	Angular gyrus	39	44, −52, 36	39, −54, 32	13		YA other > YA self
Parietal	Right	Angular gyrus	39	56, −62, 24	50, −62, 21	31		n.s.
Parietal	Right	Inferior parietal lobule	40	46, −38,32	41, −40, 30	24		OA self > OA other
Parietal	Right	Paracentral lobule	5	14, −46, 66	11, −50, 59	16		n.s.
Parietal	Left	Postcentral gyrus	2	−42, −28, 64	−41, −33, 58	42	Yes	YA other > YA self
Parietal	Left	Precuneus	7	0, −50, 64	−2, −54, 57	53	Yes	YA other > YA self
Parietal	Right	Precuneus	31	4, −72, 44	2, −73, 37	61	Yes	YA other > YA self
Parietal	Right	Precuneus	7	4, −60, 34	2, −61, 29	34		YA other > YA self
Parietal	Right	Superior parietal lobule, inferior parietal lobule	7/40	38, −64, 54	33, −66, 47	110	Yes	YA other > YA self
Parietal	Left	Superior parietal lobule, inferior parietal lobule precuneus	7	−32, −54, 62	−31, −57, 54	91	Yes	YA other > YA self
Occipital	Right	Lingual gyrus	18	26, −48, −2	23, −46, −2	41	Yes	YA other > YA self
Occipital	Right	Lingual gyrus cuneus	17/18/19/30	12, −76, −2	10, −72, −4	945	Yes	YA other > YA self
Other	Left	Caudate nucleus putamen	N/A	−20, 6, 16	−20, 3, 19	85	Yes	YA other > YA self
Other	Left	Cerebellum	N/A	−40, −64, −20	−38, −59, −20	22		OA self > OA other
Other	Right	Cerebellum	N/A	36, −76, −50	33, −68, −47	10		n.s.
Other	Left	Insula	13	−30, 22, −6	−29, 19, 0	26		OA self > OA other
Other	Right	Middle cingulate gyrus	24	12, −6, 42	10, −11, 41	43	Yes	OA self > OA other
Other	Left	Middle cingulate gyrus	24	−12, −14, 40	−13, −18, 38	14		n.s.
Other	Left	Posterior cingulate cortex	29	−10, −42, 8	−10, −42, 7	14		n.s.
Other	Right	Posterior cingulate cortex	29	4, −42, 22	2, −43, 20	27		n.s.
Other	Right	Putamen	N/A	24, −4, 6	21, −6, 9	27		YA other > YA self
Other	Left	Thalamus	N/A	−8, −12, 12	−9, −14, 14	86	Yes	OA self > OA other; YA other > YA self
Other	Right	Thalamus	N/A	22, −20, 8	19, −21, 10	159	Yes	OA self > OA other; YA other > YA self
Other	Left	Thalamus	N/A	−4, −12, −6	−5, −12, −2	90	Yes	YA other > YA self
Other	Left	Thalamus	N/A	−18, −30, 8	−18, −30, 8	17		OA self > OA other

Note: n.s. in the ‘Direction’ column indicates significant clusters for the overall self/other × age interaction that were not significant in the contrasts of interest (i.e. OA self > OA other; YA other > YA self)

Unlike the emotion/neutral ANOVA, many additional regions showed a significant self-referencing-by-age interaction. All of these regions showed the same direction of interaction (greater self > other effect for older adults than younger adults): Older adults engaged the left dorsomedial prefrontal cortex, right middle cingulate, left middle frontal gyrus, right superior and middle frontal gyri, the right middle temporal gyrus and bilateral thalamus more for self > other information. In contrast, younger adults engaged the right lingual gyrus and cuneus, bilateral superior parietal lobules, bilateral precuneus, right paracentral lobule, right postcentral gyrus, bilateral superior temporal gyri, right middle temporal gyrus, left inferior temporal gyrus, right precentral gyrus, right middle frontal gyrus, left inferior frontal gyrus, left caudate and bilateral thalamus for other > self information.

#### Subsequent memory effects

**Fig. 5:**
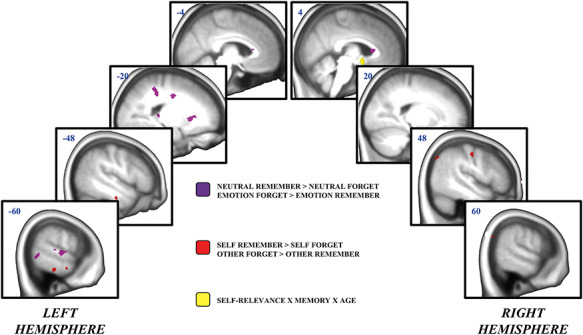
Subsequent memory effects for socioemotional information. All clusters represent activity at the whole-brain group level. Activity associated with the emotion-by-memory interaction is depicted in purple. Activity associated with the self-relevance by memory interaction is depicted in red. The three-way interaction between self-relevance, memory and age group is depicted in yellow.

Both ANOVAs revealed regions that showed main effects of memory, with the patterns generally replicating past research: Portions of the default mode network corresponded with subsequently forgotten information and large swaths of lateral prefrontal, lateral temporal and lateral occipital regions supported subsequent remembering (Supplementary Figure S2). Activation patterns were also influenced by interactions of memory-by-emotion and memory-by-self-referencing (Figure 5 and Table 5). Interestingly, the memory-by-emotion interaction arose because of regions that corresponded more strongly with subsequent remembering of neutral compared to emotional information: There was a subsequent memory effect for neutral information within the left anterior cingulate, right paracentral lobule, left inferior temporal gyrus, the left caudate and left cerebellum. In contrast, no significant clusters emerged for the subsequent memory for emotional information (emotional remembered > emotional forgotten; but see Supplementary Figure S8 for results at a reduced threshold) and only one significant cluster for subsequent forgetting, in the left superior temporal gyrus.

**Table 5:** Group-level coordinates for subsequent memory effects by condition

Lobe	Hemisphere	Region	BA	MNI (x, y, z)	Tal (x, y, z)	Cluster extent	*P* < 0.005, *k* = 40	Direction
Emotion/neutral × remember/forget								
Frontal	Right	Paracentral lobule	5	12, −28, 54	9, −33, 50	65	Yes	Neutral remember > neutral forget
Temporal	Right	Fusiform gyrus	37	52, −48, −14	47, −46, −12	15		Neutral remember > neutral forget
Temporal	Right	Hippocampus	27	38, −24, −12	34, −23, −8	24		Neutral remember > neutral forget
Temporal	Left	Hippocampus	27	−32, −32, −2	−31, −31, −1	10		n.s.
Temporal	Left	Hippocampus thalamus	30	−16, −34, 6	−16, −34, 6	55	Yes	Neutral remember > neutral forget
Temporal	Left	Inferior temporal gyrus	37	−58, −56, −2	−55, −53, −4	44	Yes	Neutral remember > neutral forget
Temporal	Left	Middle temporal gyrus	21	−40, −52, 10	−38, −51, 8	39		Neutral remember > neutral forget
Temporal	Left	Superior temporal gyrus	22/42	−62, −16, 2	−58, −17, 4	151	Yes	Emotion forget > emotion remember
Temporal	Right	Superior temporal gyrus	22	62, 0, −4	56, −2, 1	23		Emotion forget > emotion remember
Parietal	Right	Supramarginal gyrus	40	42, −30, 26	37, −32, 25	19		Emotion forget > emotion remember
Other	Left	Anterior cingulate	24	−20, −10, 40	−20,−14, 39	41	Yes	Neutral remember > neutral forget
Other	Right	Anterior cingulate	24	16, 8, 48	13, 1, 48	12		n.s.
Other	Left	Caudate putamen	N/A	−14, 26, 4	−14, 22, 10	339	Yes	Neutral remember > neutral forget
Other	Left	Cerebellum	N/A	−16, −58, −18	−16, −54, −17	53	Yes	Neutral remember > neutral forget
Other	Right	Cerebellum	N/A	4, −56, −10	3, −53, −10	39		n.s.
Other	Left	Insula	13	−34, 4, 14	−33, 1, 16	23		Emotion forget > emotion remember
Other	Left	Putamen	N/A	−24, −4, −4	−23, −5, 0	19		Neutral remember > neutral forget
Self/other × remember/forget								
Frontal	Left	Inferior frontal gyrus	34	−22, 6, −18	−21, 6, −12	11		Self remembered > self forgotten
Temporal	Right	Amygdala	N/A	22, 4, −20	20, 4, −13	28		Self remembered > self forgotten
Temporal	Right	Hippocampus	28	24, −12, −12	21, −12, −7	14		Self remembered > self forgotten
Temporal	Left	Inferior temporal gyrus	20	−58, −28, −26	−54, −25, −22	46	Yes	Self remembered > self forgotten
Temporal	Right	Middle temporal gyrus	21	70, −20, −10	64, −20, −6	53	Yes	Other forgotten > other remembered
Temporal	Left	Middle temporal gyrus	21	−58, −8, −24	−54, −7, −19	48	Yes	Self remembered > self forgotten
Temporal	Left	Parahippocampal	35	−22, −34, −4	−21, −33, −3	13		Self remembered > self forgotten
Parietal	Right	Postcentral gyrus	3	42, −18, 44	37,−23, 42	46	Yes	n.s.
Other	Left	Anterior cingulate	33	−2, 26, −8	−3, 23, −1	10		n.s.
Other	Left	Anterior cingulate	32	−6, 48, −4	−6, 43, 5	30		Other forgotten > other remembered
Other	Right	Basal ganglia	N/A	20, 2, −6	18, 1, −1	25		n.s.
Other	Left	Basal ganglia	N/A	−16, −2, −10	−16, −3, −5	32		Self remembered > self forgotten
Other	Left	Insula	13	−26, 16, −16	−25, 15, −9	26		Self remembered > self forgotten
Other	Right	Insula	13	30, 18, −14	27, 16, −6	10		Other forgotten > other remembered
Other	Right	Pons	N/A	8, −34, −28	7, −31, −24	12		n.s.
Other	Right	Pons	N/A	8, −22, −20	7, −21, −16	27		Other forgotten > other remembered

Note: n.s. in the ‘Direction’ column indicates significant clusters for the overall self/other × remember/forget interaction that were not significant in the contrasts of interest (i.e. self remembered > self forgotten; other forgotten > other remembered).

The memory-by-self-referencing interaction arose because of a stronger subsequent memory effect for self-referential than non-self-referential stimuli. In particular, there was a subsequent memory effect for self-referential stimuli (self remembered > self forgotten), with significant clusters in the left middle and inferior temporal gyri but no subsequent memory effect for non-self-referential items (other remembered > other forgotten). Instead, there was a subsequent forgetting effect for the non-self-referential condition (other forgotten > other remembered), which included a significant cluster in the right middle temporal gyrus.

To compare activity associated with the processing of emotional and self-referential content, the main effects contrast maps for emotion (emotion > neutral) and self-referencing (self > other) were overlaid. The only spatial overlap occurred in the right thalamus. To compare the subsequent memory effects for emotion and for self-referencing, the memory-by-emotion and memory-by-self interaction contrast maps were overlaid. There was no spatial overlap between the two interaction contrasts.

Finally, there were no three-way interactions between memory, emotion and age, but there was a significant interaction between memory, self-referencing and age only in the hypothalamus [MNI: *x* = 4, *y* = 2, *z* = −10]. This region appears to be engaged in the service of increased forgetting of non-self-referential information in older adults only (Supplementary Figure S3).

## Discussion

Our results provide two key insights into how emotional and self-referential information are processed and successfully encoded in older and younger adults. First, there was remarkable age similarity in the neural structures supporting the processing of emotional information yet prominent age divergence in the neural structures supporting the prioritization of self-referential information. Second, contrary to our hypothesis, enhanced encoding of emotional and self-referential information appears to arise via distinct neural mechanisms.

The effects for emotional information were largely consistent with our hypotheses insofar as older and younger adults showed similar emotional memory benefits and similar patterns of activation along the ventral visual stream during the processing of emotional information. Consistent with previous literature, both groups also showed increased activity in the lateral orbitofrontal cortex, precuneus and thalamus when processing emotional content. However, it was surprising that we did not find strong evidence for the engagement of these regions during successful *encoding* of emotional over neutral information. It also was surprising that we did not see mPFC or amygdala activity during the processing or encoding of emotional content, as activity in these regions is typically evoked by similar stimuli (Kensinger and Schacter, 2006; Kensinger *et al*., 2011). This may have reflected limitations in the paradigm, as we discuss later.

The effects of self-referencing were less consistent with our hypotheses. Self-referencing enhanced memory for both younger and older adults, but unlike emotional information, there were age-related differences in the neural mechanisms devoted to the processing of self-referential information. Older adults engaged more prefrontal regions than younger adults, including the superior and middle frontal gyrus, dorsomedial prefrontal cortex and mid-cingulate gyrus. As these regions are typically associated with a larger network engaged during self-processing (Denny *et al*., 2012; Legrand and Ruby, 2009; Qin *et al*., 2013; Beer and Flagan, 2015), it is possible these results reflect stronger self-schemas in older adults compared to younger adults. These age differences did not extend to the encoding of self-referential information into memory; the hypothalamus was the only region that differentially predicted the success of encoding self-referential information into memory with age. Although in line with prior studies showing substantial overlap in the neural regions engaged by younger and older adults during the encoding of self-referential information (Gutchess *et al*., 2015), it is somewhat surprising that the age groups did not similarly recruit neural regions during self-referential judgments (as in Gutchess *et al*., 2007). These age differences in judgment may reflect the possibility that younger and older adults approached the task differently. Indeed, a common concern in the social literature is that age differences may reflect changes in strategy rather than in the ability to engage the relevant processes (e.g. thinking of the self in a more relative and context-dependent manner with age) (Gutchess and Samanez-Larkin, 2019).

It was also surprising that our subsequent memory effects for self-referential content only showed activation in the left middle and inferior temporal gyri. These regions have been associated with successful encoding of self-referential content in previous work, yet the literature typically demonstrates increased activation in the mPFC and other cortical midline structures during the successful encoding of self-referential content across a range of experimental paradigms (Macrae *et al*., 2004; Turk *et al*., 2011; Kim and Johnson, 2014; Morel *et al*., 2014; Kalenzaga *et al*., 2015). It may be that some manipulations, such as the one used here, are sufficient to lead to a behavioral memory benefit from self-referencing, but do so without engaging the same circuitry as other self-referencing manipulations. This variability suggests a need for more diverse experimental paradigms when evaluating the encoding mechanisms supporting memory enhancements for self-referential content.

If emotional and self-referential information are indeed supported by overlapping mechanisms, we would have expected to see memory enhancements from self-referencing and emotion that were interactive and sub-additive; that is, both self-referencing and emotional valence could improve memory, but there would not be any additional benefit from combining both conditions together (Gutchess *et al*., 2007; Glisky and Marquine, 2009; Yang *et al*., 2012). Such a finding would be in line with prior ERP findings in younger adults (Fields and Kuperberg, 2012). Contrary to our hypothesis, we did not find evidence for a shared mechanism during encoding with the present paradigm. Behaviorally, emotion and self-referencing enhanced memory, with no interaction. There also was no overlapping neural activation supporting enhanced encoding of emotional and self-referential information, even when contrasts from each fMRI model were overlaid in the same space. It is important to note, however, that our paradigm elicited weak subsequent memory effects for both emotion and self-referencing. With weak findings for the individual effect of emotion or self-referencing, it becomes difficult to interpret a lack of overlap between the two. However, even when the threshold was reduced to reveal more unreliable subsequent memory effects for emotion and self-referencing individually, there was still little overlap in the regions engaged for the two (Supplementary Figure S8a). These findings suggest the need to test an alternate hypothesis, which is that while emotion and self-referencing may enhance memory via mechanisms that are broadly similar (e.g. same general anatomical regions), the precise regions of activation may differ for the two. Despite these findings, it still remains possible that an overlapping neural mechanism supports memory consolidation or retrieval of self-referential and emotional information. It may be the case that a shared mechanism emerges at a later stage of memory. Future work should consider how neural activity during memory consolidation or retrieval relates to memory success for these two categories of information.

It is also possible that our paradigm contributed to some of the unexpected findings. This novel paradigm offers the strength of more cleanly separating emotion and self-referencing. Many studies investigating self-referencing at encoding require participants to determine if personality traits describe the self or other (Gutchess *et al*., 2007; Glisky and Marquine, 2009; Yang *et al*., 2012). Trait stimuli make it difficult to differentiate the effects of self-referencing and emotion, because they can be emotionally valenced or socially desireable (i.e. ‘generous’) or undesireable (i.e. ‘mean’). Here, we were able to separately manipulate emotion, via the type of object, and self-referencing, via the condition to which the objects were assigned. Although this design allowed us to separate the effects of emotion and self-referencing within the same paradigm, this advantage may have also contributed to differences in the ways that emotional and self-referential content were processed by participants. It is possible that we found minimal overlap for the processing and successful encoding of emotional and self-referential content because these conditions were manipulated in different ways. Participants were explicitly instructed to engage with the stimuli from self or other referential perspectives, likely requiring the engagement of top-down processes, whereas the intrinsic emotional content of the objects may have relied more on bottom-up processing. We could not evaluate the basis of this potential confound in the current design, but future work should attempt to separate the effects of self-referencing and emotion while simultaneously manipulating these conditions in similar ways.

Despite the strengths of the paradigm, the design could have weakened the manipulation of emotion or self-referencing. It is possible that imagining an object in one’s home or yard, *vs* a stranger’s, was not a robust manipulation compared to previous tasks. Although previous work has shown self-referential memory enhancements when people are assigned ownership of objects (Cunningham *et al*., 2008, 2011), it is possible that this manipulation was not as robust for some of the emotional items used in this study or for older adults. A related critique of our paradigm is that, while we selected an amount of time that would be sufficient for participants to process the instructions and form relevant associations (i.e. ‘this object belongs to me, in my home/yard’ or ‘this object belongs to someone else, in their house/yard’), the trial time was insufficient to allow participants to create a detailed mental image of an object in their own home or in a novel location. One approach to address these concerns in future work may be to present participants with different objects and different situations and ask them to construct scenes from self-referential and non-self-referential perspectives. This may provide contextual salience similar to the various vingettes used in previous studies (Fields and Kuperberg, 2012, 2016; Grilli *et al*., 2018). Despite these limitations, however, both older and younger adults did show behavioral and neural self-referencing effects, suggesting that our paradigm was sensitive enough at the group level to observe information prioritization and successful encoding of the stimuli. This is consistent with recent theory proposing that the self is not only prioritized for encoding, but is a particularly salient construct that can be used to harness attention to environmental stimuli at relatively short presentation durations (Cunningham, 2016; Humphreys and Sui, 2016; Cunningham and Turk, 2017).

Overall, this was the first fMRI investigation into age differences in the processing and successful encoding of both emotional and self-referential information in a paradigm that separates the two processes. Although behavioral memory enhancements from emotion and self-referencing did not differ with age, the effects of age on socioemotional processes varied across specific domains of social and emotional processing. In contrast to the age similarity in neural activity during processing and encoding of emotional information, age differences emerged in self-referential processes. Critically, the results inform whether a single shared mechanism supports both emotional and self-referential processing, suggesting that these processes can be distinct, despite similarities suggested by prior work.

## Supplementary Material

SCAN_15_4_405_s0
