# Supplementary material for: Neural mechanisms supporting emotional and self-referential information processing and encoding in older and younger adults
Source: Soc Cogn Affect Neurosci. 2020 Apr 17;15(4):405–21. doi: 10.1093/scan/nsaa052 (PMC8561439; doi:10.1093/scan/nsaa052)
Supplement: SCAN_15_4_405_s0 [file SCAN_15_4_405_s0.docx]

**Supplementary Text**

**Methods**

**Participants.** Younger adults (n = 65; 34 female) and older adults (n = 54; 34 female) recruited from the greater Boston area enrolled in a two-part study, including one MRI session and one ERP session completed on separate days. Only data from the MRI session are reported in the manuscript. Data were completely excluded from 6 younger adults for the following reasons: Did not follow task instructions (2 males); withdrew prior to data collection (2 males; 1 female); technical problems during the task (1 male). Data were completely excluded from 7 older adults for the following reasons: withdrew during the MRI portion of the study (1 female); withdrew prior to MRI portion of study (3 females); claustrophobia (1 male; 1 female); head could not fit in the head coil (1 male). Additionally, MRI data from 14 younger adults were excluded for: Anatomical abnormality (1 male); excessive movement in the scanner (5 females, 2 males); technical problem during the task (1 male); did not have enough trials in each condition to be included in analyses (5 females). Ten older adults were also excluded from the MRI analyses for: Anatomical abnormality (1 male); excessive movement in the scanner (2 females); technical problems during the task (1 male); did not have enough trials in each condition to be included in analyses (4 females, 2 males).

Based on exclusion criteria, participants were right handed, native English speakers, who reported no history of a learning disability, neurological or neurodegenerative disorder, mental illness, or affective disorder including anxiety or depression. Prior to entering the MRI scanner all participants were screened for contraindications, including metal implants (i.e., plates, screws, rods, pacemakers, cochlear implants), pregnancy, and history of claustrophobia.

**Materials**

**Cognitive Testing.** In order to characterize the study sample, in a separate session, participants completed cognitive tests (Table 1) from the Wechsler Adult Intelligence Scale-IV: Digit Symbol, Digit Span, Arithmetic, Mental Control, Verbal Paired Associates, and Visual Paired Associates (Wechsler, 2008). The Logical Memory sub-scale of the Wechsler Memory Scale-III was also administered (Wechsler, 1997), as was the California Verbal Learning Test-II (CVLT; Delis, Kramer, Kaplan & Ober, 1987), the F-A-S subtest of the Neurosensory Center Comprehensive Examination for Aphasia (Spreen & Benton, 1977), the Shipley Vocabulary Test (Shipley, 1986), and Digit Comparison (Hedden et al., 2002).

**Experimental Stimuli.** Normative data were taken from the published OASIS norms (68 objects) and from ratings made by a separate group of participants, across the adult lifespan, on Amazon’s Mechanical Turk (MTurk; 352 objects) using 9-point Likert scales for valence (1 = *highly negative*, 5 = *neither negative nor positive*, 9 = *highly positive*), arousal (1 = *highly calming or subduing*, 9 = *highly arousing or agitating*), and self-relevance (1 = *low self-relevance*, 9 = *high self-relevance*).

After task completion, study participants also rated the images for valence and arousal (they did not rate self-relevance as that had been manipulated at encoding). Valence ratings were subjected to a repeated measures ANOVA with emotion (negative, neutral, positive) as a within-subjects factor and age (older adults and younger adults) as a between-subjects factor. Mauchly’s test for sphericity was violated χ^2^(2) = .23, *p* < .001 and degrees of freedom were corrected using Greenhouse-Geisser estimates (ε = .57). This analysis revealed a significant main effect of emotional valence, *F*(1.13, 117.65) = 467.71, *p* < .001, ${}_{G}^{2}$= .694 (Olejnik & Algina, 2003). Paired samples t-tests revealed that negative objects were rated as significantly more negative than neutral objects, *t*(105) = 19.70, *p* < .001, *d_av_* = 2.08 (Note: For within-subject pairwise comparisons, *d_av_* is the measure of effect size whereby the difference of the means for each condition is divided by their pooled standard deviation; Cumming, 2013; Lakens, 2013). Similarly, positive objects were rated as significantly more positive than neutral, *t*(105) = 21.60, *p* < .001, *d_av_* = 1.87. Emotional valence did not interact with age, *F*(1.13, 117.65) = .30, *p* = .744, ${}_{G}^{2}$= -.003. However, there was a significant main effect of age [*F*(1, 104) = 6.54, *p* = .01, ${}_{G}^{2}$= .025], such that older adults, on average, rated all objects as more positive than younger adults.

Arousal ratings were submitted to a repeated measures ANOVA with emotion (negative, neutral, and positive) as a within-subject factor and age (older adults and younger adults) as a between-subjects factor. Mauchly’s test for sphericity was violated χ^2^(2) = .47, *p* < .001 and degrees of freedom were corrected using Greenhouse-Geisser estimates (ε = .65). This analysis revealed a significant main effect of emotion, *F*(1.31, 135.98) = 22.10, *p* < .001, ${}_{G}^{2}$= .082. Paired samples *t*-tests revealed that negative objects were rated as more arousing than neutral objects [*t*(105) = 9.32, *p* < .001, *d_av_* = .98], and positive objects were also rated as more arousing than neutral objects [*t*(105) = 5.12, *p* < .001, *d_av_* = .47]. Importantly, positive and negative objects were rated as equally arousing *t*(105) = 1.52, *p* = .13, *d_av_* = .20. Emotional valence did not interact with age for arousal ratings [*F*(1.31, 134.98) = 1.27, *p* = .27, ${}_{G}^{2}$ = .001], and there was no main effect of age [*F*(1, 104) = .16, *p* = .69, ${}_{G}^{2}$= -.004].

**Anatomical Image Acquisition.** Whole-brain anatomical images were acquired with a single-shot interleaved multi-slice T1-weighted structural scan (3D MEMPRAGE, Sagittal Slices = 176, Slice Thickness = 1mm, Voxel Size = 1mm^3^, FOV = 256mm, TR = 2530ms, TE1 = 1.69ms, TE2 = 3.55ms, TE3 = 5.41ms, TE4 = 7.27ms, Flip Angle = 7°).

**Valence interactions with age and self-referencing**

It is well established that the emotional valence of information can differentially impact memory encoding success in older and younger adults. Older adults are known to have a bias towards positive information (Reed et al., 2014). This positivity bias is thought to be driven by a shift in motivation to attend to more positive information as we age, rather than age-related deterioration in cognition (Mather & Carstensen, 2005; Reed & Carstensen, 2012). Alongside better memory for positive information, older adults exhibit greater activity in the medial prefrontal cortex when processing positive compared to negative information (Leclerc & Kensinger, 2008), and the medial prefrontal cortex and amygdala are known to modulate activity in the hippocampus during the successful encoding of positive information (Addis et al., 2010). Self-referential information is also known to interact with emotional valence. Generally, individuals tend to rate themselves as more positive than others (Alicke & Govorun, 2005). As a result, positive information tends to be remembered to a greater extent than negative information from self-referential perspectives (Mezulis et al., 2004).

In the present study, we did not report on behavior or neural activity for valence by age or valence by self-referencing interactions. However, we ran a repeated measures ANOVA using d-prime scores for “Remembered” items, with valence (positive, negative, neutral) and referencing (self and other) as within-subjects factors, and age group (older adults and younger adults) as between-subjects factors (Supplementary Figure 4). There was a main effect of emotional valence, *F*(2, 208) = 15.67, *p* < .001, ${}_{G}^{2}$= .015. Follow-up paired samples *t*-tests revealed that participants had better memory for negative compared to neutral objects [*t*(105) = 4.72, *p* < .001, *d*_av_ = .29] and positive compared to neutral objects [*t*(105) = 4.46, *p* < .001, *d*_av_ = .25]. There was also a main effect of self-referencing [*F*(1, 104) = 32.29, *p* < .001, ${}_{G}^{2}$= .008], such that across groups, memory for objects was better in the self than the other condition. Similar to the results in the main paper, there was no main effect of age [*F*(1, 104) = .02, *p* = .88, ${}_{G}^{2}$= -.008], and age did not interact with emotional valence [*F*(2, 208) = 2.90, *p* = .06, ${}_{G}^{2}$= .002] or self-referencing[*F*(1, 104) = .70, *p* = .41, ${}_{G}^{2}$= .000]. Thus, young and older adults’ performance on the task, and their memory benefits from emotion and self-referencing, did not significantly differ. Emotional valence did not interact with self-referencing [*F*(2, 208) = .51, *p* = .60, ${}_{G}^{2}$= .000], and there was no three-way interaction between emotional valence, self-referencing and age [*F*(2, 208) = 1.92, *p* = .15, ${}_{G}^{2}$= .000]. As with the results reported in the paper, a separate repeated measures ANOVA was run on “Know” responses. This analysis only revealed a significant main effect of self-referencing [*F*(1, 104) = 7.20, *p* = .008, ${}_{G}^{2}$ = .008] , such that across groups, and similar to the results reported in the paper, memory for objects was better in the Other condition than the Self condition (Supplementary Figure 5).

Even though there was no emotional valence by age interaction for memory performance, we suspected there may have been age differences in the way that positive and negative information are processed and successfully encoded. Similar to the Emotion-Neutral and Self-Other models reported in the main paper, we created a “Positive-Negative” general linear model to examine the neural activity during the processing and encoding of positive and negative information. Each participant’s data was first subjected to a fixed effects model that collapsed across self-referential conditions and consisted of regressors for positive-remembered, positive-forgotten, negative-remembered, and negative forgotten. Similar to the other two models, all “known” objects were entered as one regressor (which was not brought to group level analyses), and a linear drift regressor was also included. The results were then subjected to a random effects ANOVA using positive/negative and remembered/forgotten as within-subject variables and age as a between subject variable. This ANOVA will be referred to as the “Positive-Negative” ANOVA. When significant interactions were revealed in the ANOVA, post-hoc analyses were conducted to determine the direction of the interaction.

The Positive-Negative ANOVA, revealed clusters that showed an age-invariant significant main effect of emotion (Supplementary Figure 6). Directional *t*-tests revealed that across older and younger adults, the left inferior temporal gyrus, and left precentral gyrus were engaged for negative > positive information. There were no clusters that reached significance identified by the positive > negative *t*-test. There was also a significant valence by memory interaction. There was a subsequent memory effect for negative information (negative-remember > negative-forgotten), which included significant clusters in the left BA6, BA10, BA11, BA21, BA38, BA44, BA47and and bilateral BA8 and cerebellum (Supplementary Figure 7). By contrast, there were no significant clusters revealed for the subsequent memory for positive information (positive-remembered > positive forgotten).

None of the results from this model provided any insight into age differences during the processing of emotional valence. We did not see any age by valence interactions. As a result, this model was not included in the main body of this paper. It should also be noted that while we were able to examine the interaction between emotional valence and self-referencing behaviorally, this was not possible with the fMRI data. Too many participants would need to be removed from the sample to ensure that each participant had enough trials per bin size. The results in this case would be uninterpretable due to lack of power.

**
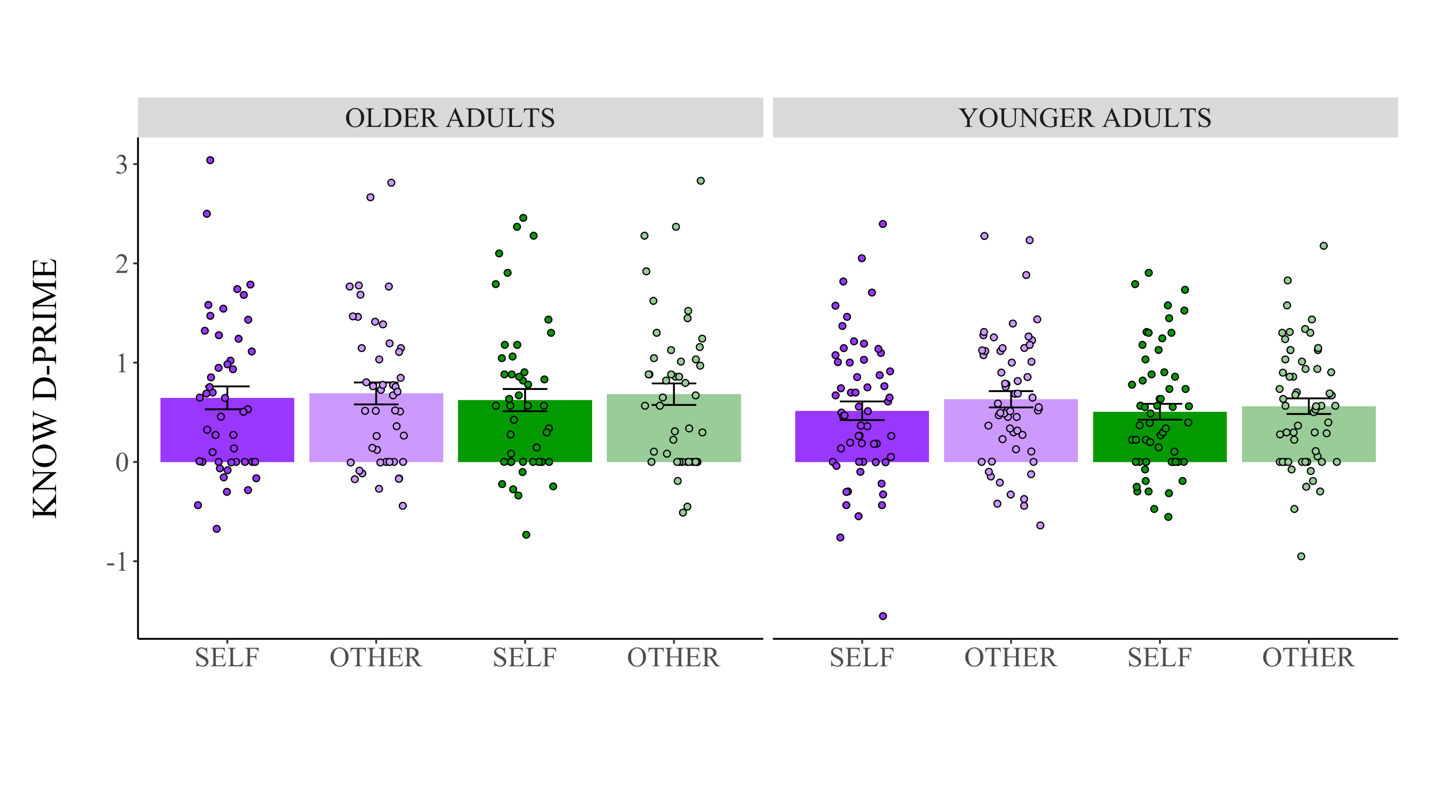
**

***Supplementary Figure 1*. Behavioral Memory Performance for Items Indicated as “Know”.** Error bars represent ± SEM. Performance on emotion trials are indicated in purple, while neutral trials are indicated in green. A repeated measures ANOVA revealed a significant main effect of self-relevance [*F*(1, 104) = 6.36, *p* = .01, ${}_{G}^{2}$ = .002], such that both groups had better memory for objects in the Other condition (light bars) compared to objects in the Self condition (dark bars).

**
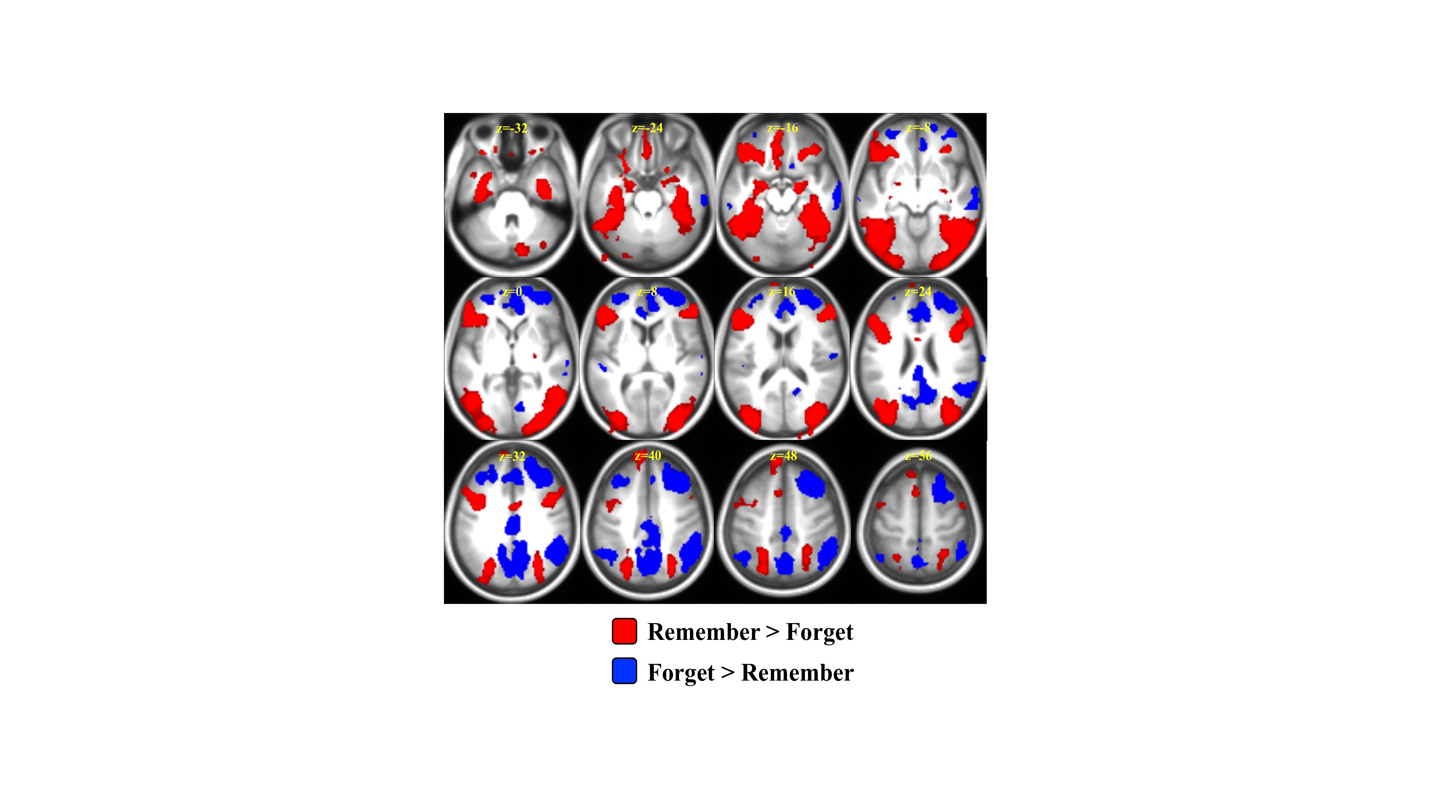
**

***Supplementary Figure 2.* Main effect of memory across Emotion-Neutral ANOVA and Self-Other ANOVA.** To produce the ‘Remember > Forget’ (red) contrast a conjunction analysis was conducted whereby the ‘Remember > Forget’ contrast for the Self-Other ANOVA was inclusively masked with the ‘Remember > Forget’ contrast from the Emotion-Neutral ANOVA. The same method was used to create the ‘Forget > Remember’ (blue) contrast. Each contrast was thresholded at *p =* .005 with a cluster extent of *k* = 40.

**
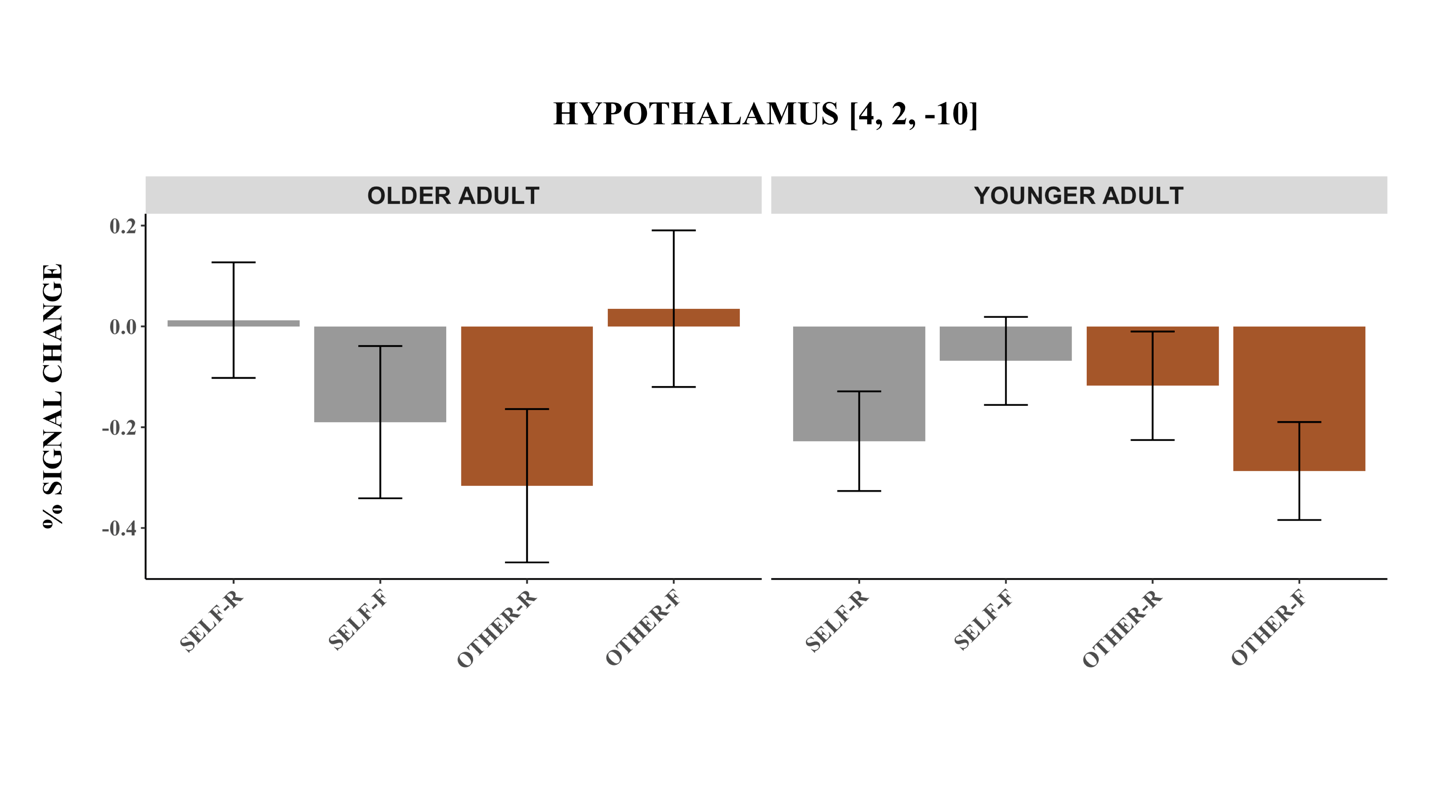
**

***Supplementary Figure 3.* Age differences in subsequent memory for self-relevant information.** Error bars indicate ± SEM. A hypothalamus ROI was created using the marsBaR toolbox in SPM12 based on the hypothalamus cluster identified in the self-relevance x age x memory interaction in the Self-Other ANOVA. Beta estimates were then pulled for this ROI using the REX toolbox for each condition from each subject’s fixed effects model. Betas were then converted to percent signal change and plotted in this figure.

**
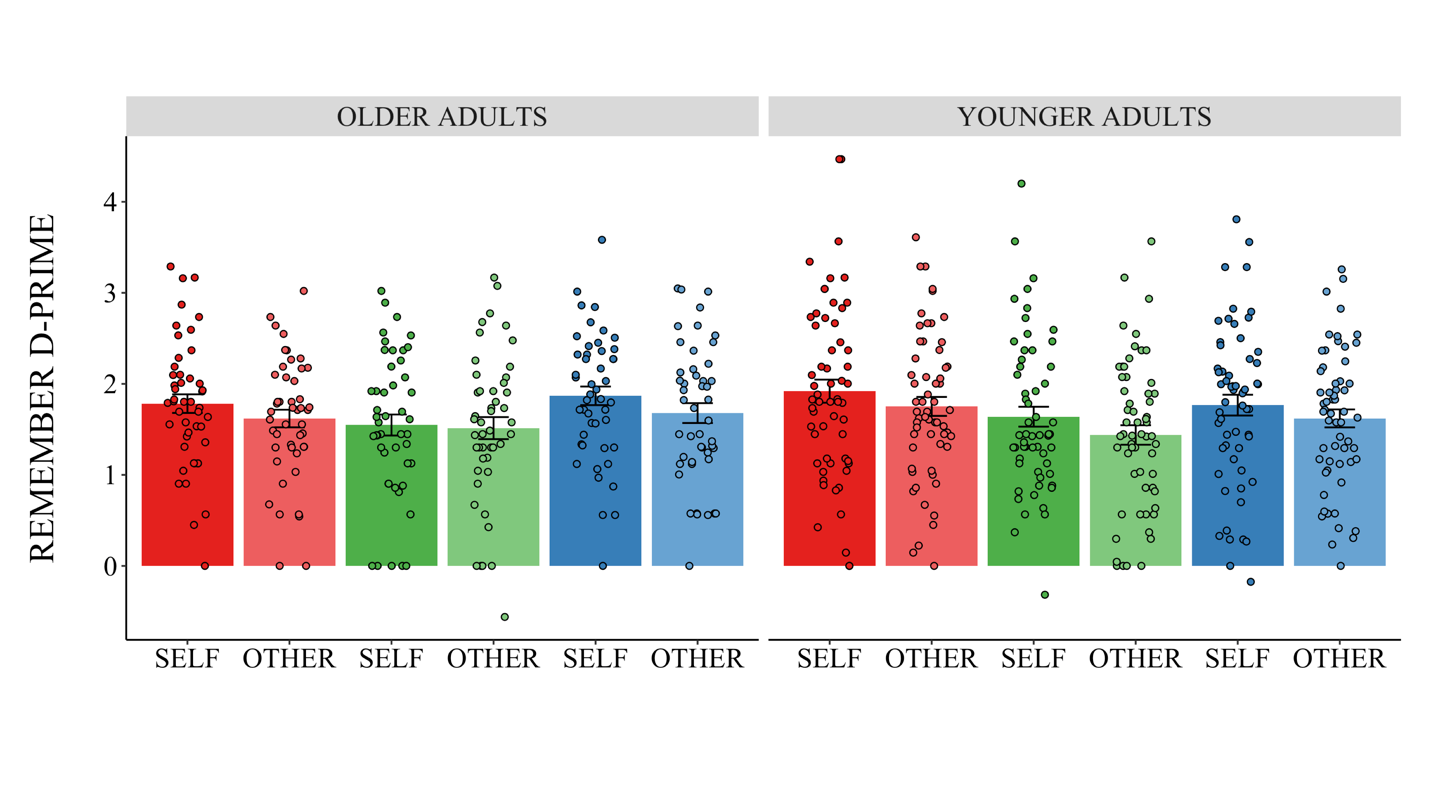
**

***Supplementary Figure 4*. Behavioral Memory Performance Across Valence for Items Indicated as “Remember”.** Error bars represent ± SEM. There was a main effect emotion. Both groups had better memory for negative objects (red bars) compared to neutral objects (green bars) and positive objects (blue bars) compared to neutral objects. There was also a main effect of self-relevance. Both groups had better memory for objects in the Self condition (dark bars) compared to objects in the Other condition(light bars).

**
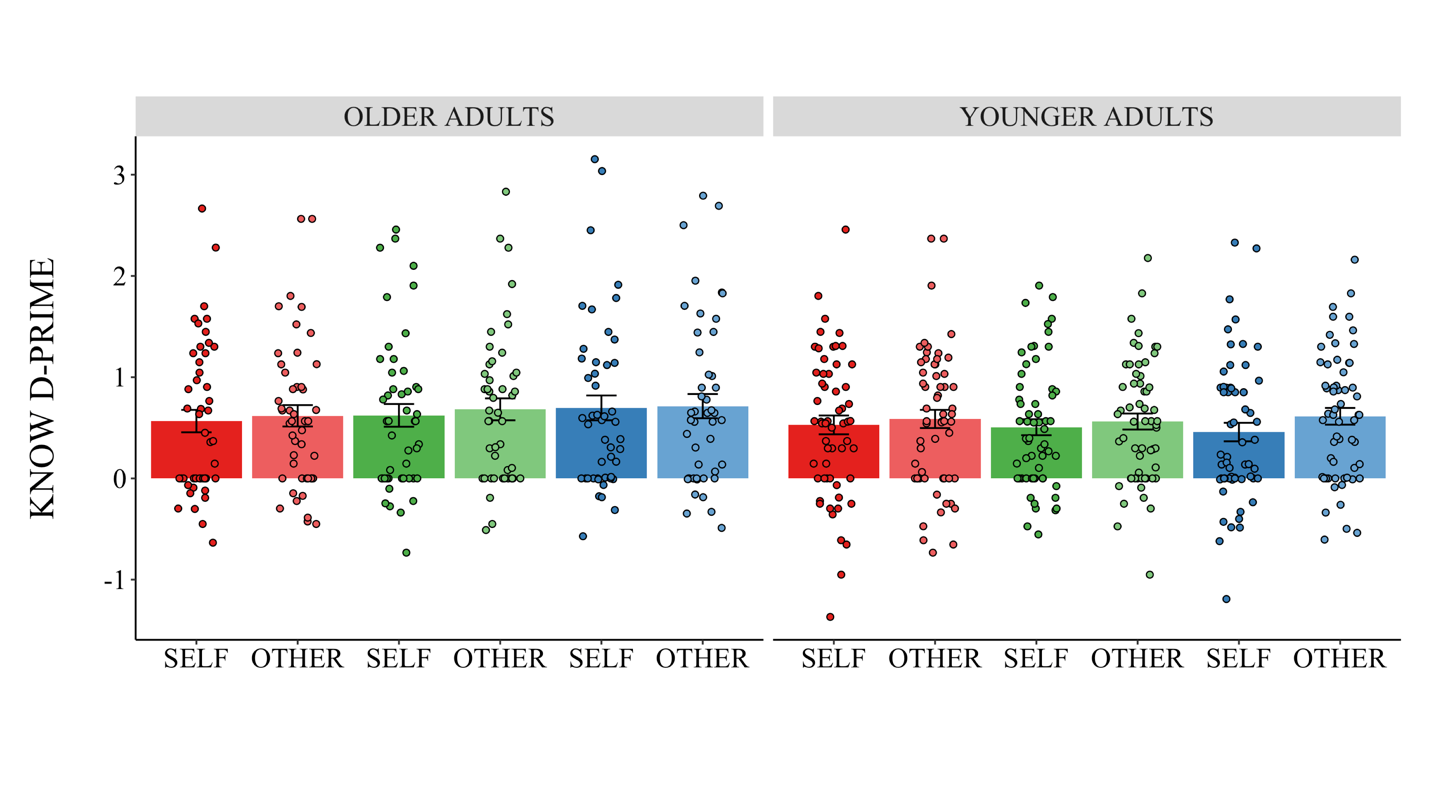
**

***Supplementary Figure 5*. Behavioral Memory Performance Across Valence for Items Indicated as “Know”.** Error bars represent ± SEM. Negative objects are indicated in red, neutral objects are indicated in green, and positive objects are indicated in blue. There was a main effect of self-relevance. Both groups had better memory for objects in the Other condition (light bars) compared to objects in the Self condition (dark bars).

**
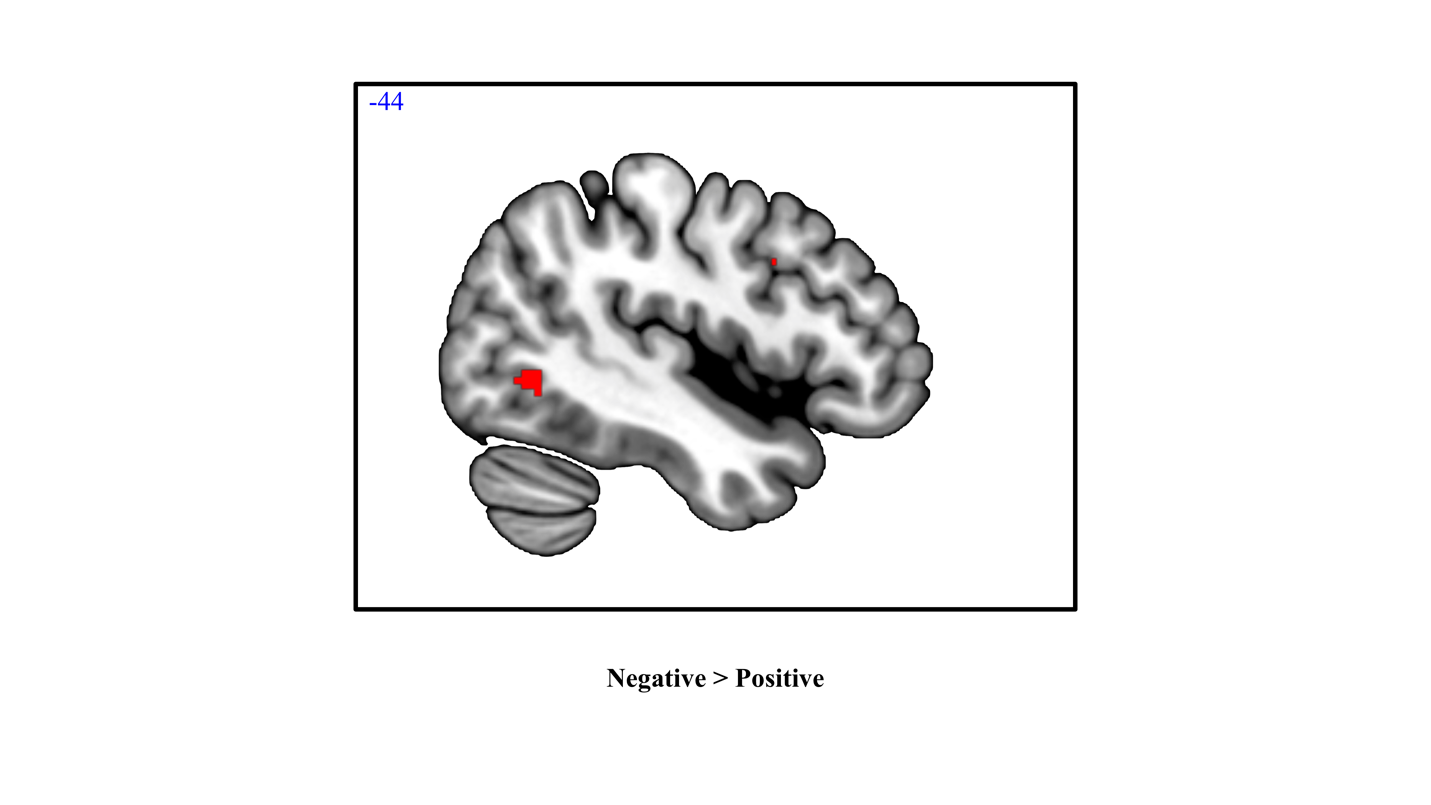
**

***Supplementary Figure 6*. Processing Emotional Valence.** All clusters represent activity at the whole brain group-level from the Positive-Negative ANOVA. Activity associated with the processing of Negative > Positive stimuli are depicted in red. This contrast was thresholded at *p* = .005 with a cluster extent of *k* = 40.

**
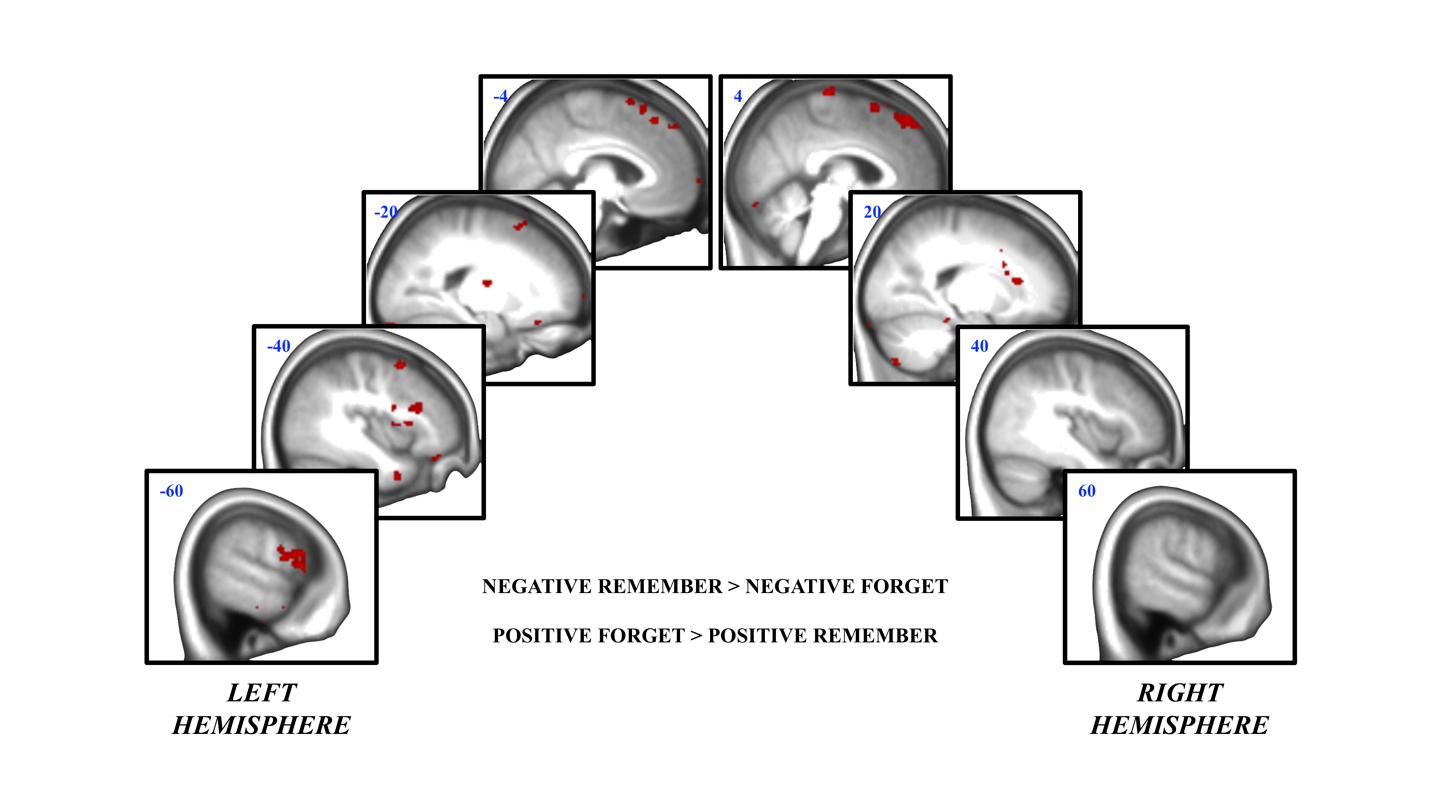
**

***Supplementary Figure 7*. Subsequent memory effects for emotional valence.** All clusters represent activity at the whole brain group-level from the Positive-Negative ANOVA. Activity associated with the valence (positive and negative) by memory interaction is depicted in red. This contrast was thresholded at *p* = .005 with a cluster extent of *k* = 40.

**
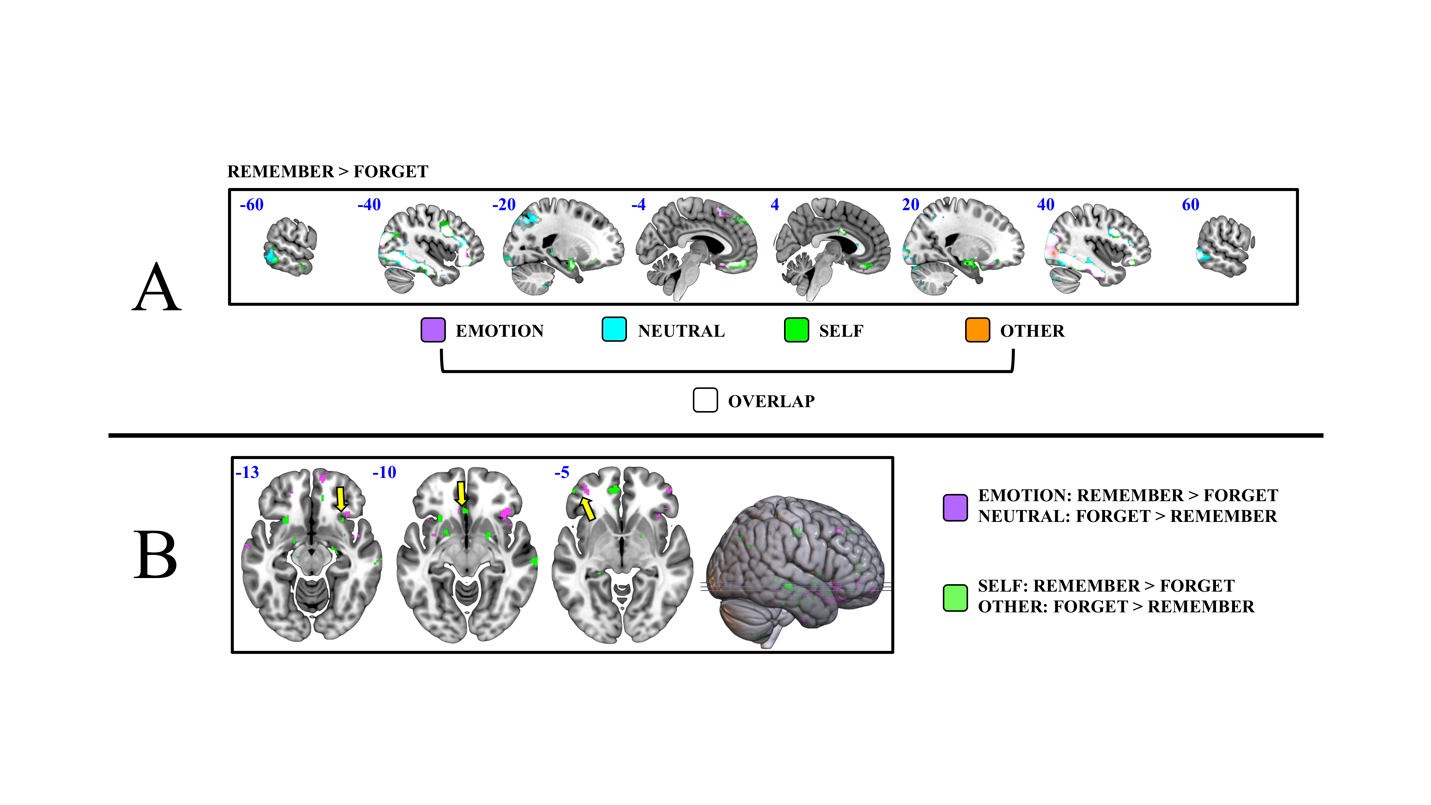
**

***Supplementary Figure 8*. Subsequent Memory Overlap for Emotion and Self-relevance at Lower Thresholds.** A: ‘Remember > Forget’ *t*-contrasts (*p* = .005, *k* = 40) were created for each condition: Emotion (purple), Neutral (cyan), Self (green), Other (orange). These *t*-contrasts were overlaid in the same space to reveal a common memory network across each condition that is consistent with previous literature. B: An emotion by memory interaction *t*-contrast (‘Emotion: Remember > Forget’, ‘Neutral Forget > Remember; *p* = .05, *k* = 25) was created for the Emotion-Neutral ANOVA (purple) and a self by memory interaction *t*-contrast (‘Self: Remember > Forget’, ‘Other: Forget > Remember’; *p* = .01, *k* = 40) was created for the Self-Other ANOVA (green). These contrasts were overlaid in the same space to reveal the same general regions of activation (yellow arrows), but very little overlap during successful encoding of emotional and self-relevant content. *Note:* Contrasts were not masked with *F-*contrasts as this figure is for visualization purposes only.

**Supplementary References**

Addis, D. R., Leclerc, C. M., Muscatell, K. A., & Kensinger, E. A. (2010). There are age-related changes in neural connectivity during the encoding of positive, but not negative, information. *Cortex*, *46*(4), 425–433. https://doi.org/10.1016/j.cortex.2009.04.011

Alicke, M. D., & Govorun, O. (2005). The Better-Than-Average Effect. In M. D. Alicke, D. A. Dunning, & J. I. Krueger (Eds.), Studies in self and identity. The Self in Social Judgment (pp. 85-106). New York, NY, US: Psychology Press.

Cumming, G. (2013). *Understanding The New Statistics: Effect Sizes, Confidence Intervals, and Meta-Analysis*. Routledge. https://doi.org/10.4324/9780203807002

Delis, D. C., Kramer, J. H., Kaplan, E., & Ober, B. A. (1987). *Manual for the California verbal learning test*. San Antonio, TX: The Psychological Corporation.

Hedden, T., Park, D. C., Nisbett, R., Ji, L.-J., Jing, Q., & Jiao, S. (2002). Cultural variation in verbal versus spatial neuropsychological function across the life span. *Neuropsychology*, *16*(1), 65–73. https://doi.org/10.1037/0894-4105.16.1.65

Lakens, D. (2013). Calculating and reporting effect sizes to facilitate cumulative science: A practical primer for t-tests and ANOVAs. *Frontiers in Psychology*, *4*. https://doi.org/10.3389/fpsyg.2013.00863

Leclerc, C. M., & Kensinger, E. A. (2008). Age-related differences in medial prefrontal activation in response to emotional images. *Cognitive, Affective, & Behavioral Neuroscience*, *8*(2), 153–164. https://doi.org/10.3758/CABN.8.2.153

Mather, M., & Carstensen, L. L. (2005). Aging and motivated cognition: The positivity effect in attention and memory. *Trends in Cognitive Sciences*, *9*(10), 496–502. https://doi.org/10.1016/j.tics.2005.08.005

Mezulis, A., Abramson, L. Y., Hyde, J. S., & Hankin, B. L. (2004). Is there a universal positivity bias in attributions? A meta-analytic review of individual, developmental, and cultural differences in the self-serving attributional bias. *Psychological Bulletin*, *130*(5), 711–747. https://doi.org/10.1037/0033-2909.130.5.711

Olejnik, S., & Algina, J. (2003). Generalized Eta and Omega Squared Statistics: Measures of Effect Size for Some Common Research Designs. *Psychological Methods*, *8*(4), 434–447. https://doi.org/10.1037/1082-989X.8.4.434

Reed, A. E., & Carstensen, L. L. (2012). The Theory Behind the Age-Related Positivity Effect. *Frontiers in Psychology*, *3*. https://doi.org/10.3389/fpsyg.2012.00339

Reed, A. E., Chan, L., & Mikels, J. A. (2014). Meta-analysis of the age-related positivity effect: Age differences in preferences for positive over negative information. *Psychology and Aging*, *29*(1), 1–15. https://doi.org/10.1037/a0035194

Shipley, W. C. (1986). *Shipley institute of living scale.* Los Angeles: Western Psychological Services.

Spreen, F. O., & Benton, A. L. (1977). *Manual of instructions for the neurosensory center comprehensive examination for aphasia*. Victoria, BC: University of Victoria.

Wechsler, D. (1997). *WAIS-3: Wechsler Adult Intelligence Scale: Administration and Scoring Manual*. New York, NY: Psychological Corporation.

Wechsler, D. (2008). *Wechsler adult intelligence scale–Fourth Edition (WAIS–IV)*. San Antonio, TX: NCS Pearson.
